# Transcriptomic Profiling of Adipose Derived Stem Cells Undergoing Osteogenesis by RNA-Seq

**DOI:** 10.1038/s41598-019-48089-1

**Published:** 2019-08-13

**Authors:** Shahensha Shaik, Elizabeth C. Martin, Daniel J. Hayes, Jeffrey M. Gimble, Ram V. Devireddy

**Affiliations:** 1 Bioengineering Laboratory, Department of Mechanical Engineering, Louisiana State University, Baton Rouge, LA USA; 2 Department of Biological and Agricultural Engineering, Louisiana State University, Baton Rouge, LA USA; 3 Department of Biomedical Engineering, Pennsylvania State University, University Park, PA USA; 4 La Cell LLC and Center for Stem Cell Research & Regenerative Medicine and Departments of Medicine, Structural & Cellular Biology, and Surgery, Tulane University School of Medicine, New Orleans, LA USA

**Keywords:** Adult stem cells, Transcriptomics, Transcriptomics, Stem cells, Stem cells

## Abstract

Adipose-derived stromal/stem cells (ASCs) are multipotent in nature that can be differentiated into various cells lineages such as adipogenic, osteogenic, and chondrogenic. The commitment of a cell to differentiate into a particular lineage is regulated by the interplay between various intracellular pathways and their resultant secretome. Similarly, the interactions of cells with the extracellular matrix (ECM) and the ECM bound growth factors instigate several signal transducing events that ultimately determine ASC differentiation. In this study, RNA-sequencing (RNA-Seq) was performed to identify the transcriptome profile of osteogenic induced ASCs to understand the associated genotype changes. Gene ontology (GO) functional annotations analysis using Database for Annotation Visualization and Integrated Discovery (DAVID) bioinformatics resources on the differentially expressed genes demonstrated the enrichment of pathways mainly associated with ECM organization and angiogenesis. We, therefore, studied the expression of genes coding for matrisome proteins (glycoproteins, collagens, proteoglycans, ECM-affiliated, regulators, and secreted factors) and ECM remodeling enzymes (MMPs, integrins, ADAMTSs) and the expression of angiogenic markers during the osteogenesis of ASCs. The upregulation of several pro-angiogenic ELR+ chemokines and other angiogenic inducers during osteogenesis indicates the potential role of the secretome from differentiating ASCs in the vascular development and its integration with the bone tissue. Furthermore, the increased expression of regulatory genes such as CTNNB1, TGBR2, JUN, FOS, GLI3, and MAPK3 involved in the WNT, TGF-β, JNK, HedgeHog and ERK1/2 pathways suggests the regulation of osteogenesis through interplay between these pathways. The RNA-Seq data was also validated by performing QPCR on selected up- and down-regulated genes (COL10A1, COL11A1, FBLN, FERMT1, FN1, FOXF1, LAMA3, LAMA4, LAMB1, IGF1, WNT10B, MMP1, MMP3, MMP16, ADAMTS6, and ADAMTS14).

## Introduction

There are two types of bone forming mechanisms- endochondral ossification and intramembranous ossification^1–3^. In endochondral ossification, the cells first differentiate into chondrocytes secreting large volumes of extracellular matrix which eventually fuse together to be replaced by bone cells^1–3^. In the case of intramembranous ossification, cells directly differentiate into bone cells, a process that occurs pre-dominantly in flat bones^1–3^. The vascularization of bone tissue is especially important for proper bone formation during endochondral ossification^1–3^. Thus, the synchronization between osteogenesis, extracellular matrix production, chondrogenesis, and angiogenesis is critical for the development, remodeling, repair and functioning of healthy bone tissue. In particular, the interactions between cells and extracellular matrix (ECM) regulates cell proliferation, differentiation, migration, and signaling^4,5^. Some of the main components of ECM are fibronectin, collagens, laminins, proteoglycans, glycoproteins, et cetera^6–8^. ECM is secreted by the cells and its composition, structure, and other material properties such as elasticity and stiffness varies from tissue to tissue that ultimately determines the stem cell fate^4–6,9^. For example, stiffer ECM generally promote osteogenesis whereas softer ECM favors chondrogenic and adipogenic differentiations^9,10^.

ECM’s regulatory ability is compromised by imbalances in ECM remodeling enzymes, ECM injury, or biological aging and senescence of the cells that deposit ECM^11^. The ECM regulation and homeostasis is accomplished by a remodeling process that involves degradation and regeneration of ECM components. Matrix metalloproteinases (MMPs) and a disintegrin along with metalloproteinase with thrombospondin motifs (ADAMTS) are the two major enzymatic families that degrade ECM whereas tissue inhibitors of metalloproteinases (TIMPs) inhibit the activity of MMPs and ADAMTSs enzymes^12^. MMPs, also called Matrixins, are a group of endopeptidases that are Ca2+ and Zn2+ dependent for proteolytic activation and play an active role to degrade collagens in ECM^13^. ADAMTSs are secreted extracellularly and cleave mainly proteoglycans, aggrecans, and pro-collagens within the ECM^12^. Integrins are heterodimeric transmembrane receptors that act as molecular bridges between the cytoplasm and the ECM to facilitate the transmission of mechano-transductory signals stimulated by binding of ECM components to the receptor portion of integrins. This interplay between the cytoplasm and ECM via the integrins regulates proliferation, cell-migration, survival, and differentiation^14^. In addition, the secretome from ASCs is known to exhibit pro-angiogenic effects by autocrine and paracrine signaling^15,16^. The secretome composition may differ in response to the cell differentiation and can influence the cell’s differentiation potential^15,16^. Furthermore, the pro-angiogenic paracrine effects of ASCs are evident when the ischemic models treated with ASCs restored the blood flow by the regeneration of vasculature^17–19^. Thus, the ECM synthesis and the secretion of secretome plays a vital role in the behavior of ASCs in response to several intrinsic and extrinsic stimuli to develop into fully functional bone.

In this article, we performed RNA-Seq analysis to compare the global gene expression of undifferentiated ASCs and osteogenic differentiated ASCs. The differentially expressed genes between these two groups were analyzed to determine the enriched pathways using the gene ontology functional analysis by DAVID bioinformatics. Based on the enriched pathways, we studied the ECM genes associated with the matrisome, ECM remodeling enzymes (MMPs, and ADAMTSs), integrins, and secretomic genes to gain insight into the regulatory roles of osteo-ECM and the secretome during osteogenesis to enhance bone formation and vascularization. Furthermore, the RNA-Seq data was analyzed to correlate and contrast the relationship between angiogenic and osteogenic genes and their transcription factors.

## Materials and Methods

### ASC cell culture

Human ASCs frozen at P0 were procured from LaCell LLC, New Orleans, LA. The vials were thawed at 37 °C in a water bath for 1–2 minutes and diluted with StromaQual™ medium supplemented with 10% FBS and 1% antibiotic dropwise to remove cryoprotectant agent. The samples were centrifuged at 300 g for 5 min and the obtained pellet was suspended in StromaQual and cultured until passage2 (P2) in a cell culture incubator at 37 °C with 5% humidified CO_2_ as described elsewhere^20–22^.

### Osteogenic differentiation

ASCs at P2 from 3 different donors were counted and pooled together with equal numbers and plated in 12 well plates at a density of 1 × 10^4^ cells/cm^2^ and cultured to at least 90% confluence. Osteogenic differentiation was carried out using stromal media supplemented with 10 mM β-glycerophosphate, 50 µg/ml L-Ascorbic acid 2-phosphate sesquimagnesium salt hydrate, and 10 nM dexamethasone for 21 days by replacing media every 3 days^21,23^. To stain the deposited calcium in extracellular matrix the cells were fixed in 70% ice cold ethanol and stained with 2% alizarin red solution (pH adjusted to 4.1–4.3).

### RNA isolation, Reverse transcription, and QPCR

RNA was isolated using the Purelink RNA kit (Life Technologies) according to manufacturer’s instructions. The quality and quantity of isolated RNA was measured using nanodrop spectrophotometer. The first strand cDNA synthesis was done by using high capacity cDNA synthesis kit (Applied Biosciences). For quantitative real time PCR sybr green (Applied Biosystems) kit was used as per manufacturer’s instructions on abi QPCR machine. Following the QPCR the relative fold change in expression was calculated 2^**−(Δ ΔCt)**^ method with GAPDH as internal reference control^24^. All the primer sequences are presented in Supplemental Table S1.

### cDNA Library preparation for RNA-sequencing

SMART-Seq v4 Ultra Low Input RNA Kit for Sequencing (Clontech, Cat. No. 634888) was used to perform cDNA synthesis using template switching technology. Prior to generating the final library for Ion Torrent sequencing, 2.5 µg of cDNA was simultaneously digested with AfaI restriction endonuclease to remove SMART adapters and enzymatically sheared using reagents from the Ion Xpress Plus Fragment Library Preparation Kit (Life Technologies, Cat. No. 4471269). Templates for RNASeq were prepared with the Ion PI™ Hi-Q OT2 200 template Kit (Life Technologies, Cat. No. A26434) using the Ion OneTouch™ 2 System for Ion Proton™ System semiconductor sequencing. These templates were sequenced using the Ion PI™ Hi-Q Sequencing 200 Kit (Life Technologies, Cat. No. A26433) and Ion PI™ Chip v3, on the Ion Proton Sequencer. Preliminary analysis of the ensuing sequencing data pertaining to quality of the run, read lengths and coverage were performed using the torrent suite software.

### Differential gene expression analysis on RNA-Seq data

The data obtained in FASTQ file format from RNA-sequencing was aligned to ensemble hg38 human genome using the STAR program. RSEM was used to determine the differential expression between undifferentiated and osteogenic differentiated ASCs. The differentiation fold change between undifferentiated and differentiated ASCs was calculated by dividing the number of counts of differentiated sample over number of counts of undifferentiated sample. The genes with the fold change (FC) above 1.5 were considered as upregulated whereas the genes with the fold change (FC) below 0.66 were treated as downregulated and are chosen for further analysis.

### Gene Ontology (GO) Functional analysis

The upregulated (above FC 1.5) and the downregulated (below FC 0.66) genes were sorted into different groups based on the fold change in gene expression. Under the upregulated category, 5 groups are made such as above 25 fold, between 24.99–10 fold, 9.99–5 fold, 4.99–2.5 fold, and 2.49–1.5 fold. Similarly, for the downregulated category, 4 groups are made such as between 0.66–0.4 fold, 0.39–0.2 fold, 0.19–0.1 fold, and less than 0.09 fold. Each group was subjected to gene ontology (GO) functional annotation analysis under biological processes category using DAVID Bioinformatics Resources (http://david.abcc.ncifcrf.gov/home.jsp) to determine significantly enriched genes^25^.

## Results

To assess the overall gene changes following osteogenic differentiation, three pooled donor ASCs were induced with osteogenic differentiation media for 21 days and next generation RNA sequencing was performed as described in the methods. Confirmation of osteogenic induction was observed through alizarin red stain and is shown in Sup Fig. S1. Briefly, positive staining was observed in osteogenic medium treated ASCs indicating osteogenic differentiation (Sup Fig. S1). After the alignment of RNA-Seq data to the human genome hg38, the expression of pro-osteogenic genes was evaluated to confirm osteognic differentiation (Sup Table S2). Most notable osteogenic genes that shown increased expression were BGLAP (Osteocalcin), COL10A1, alkaline phosphatase (ALPL), and BMP4. Visualization and confirmation of enhanced gene expression between control and osteo-induced ASCS were also evaluated through the Integrative Genome Viewer (IGV)^26^. Representative images for individual osteogenic genes are shown in (Sup Fig. S2).

**Figure 1:**
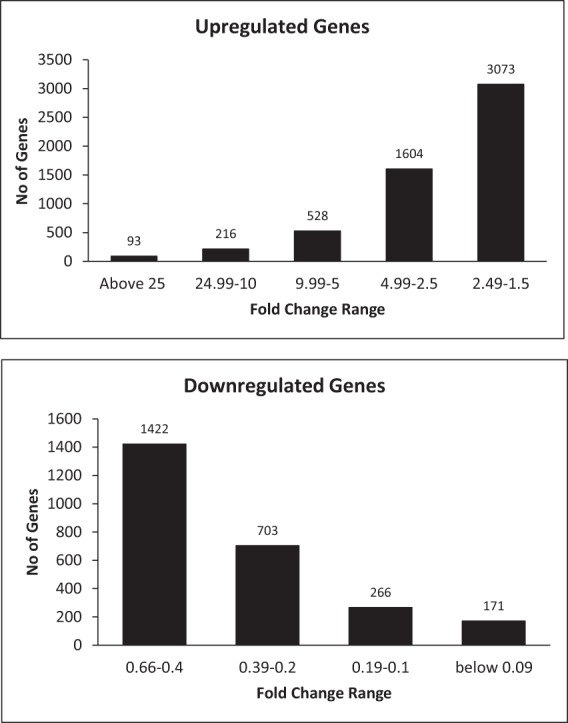
Classification of Genes Based on Expressional Fold Changes’. The genes with fold change above 1.5 considered as upregulated and less than 0.66 were categorized as downregulated.

### Gene ontology functional analysis

**Figure 2:**
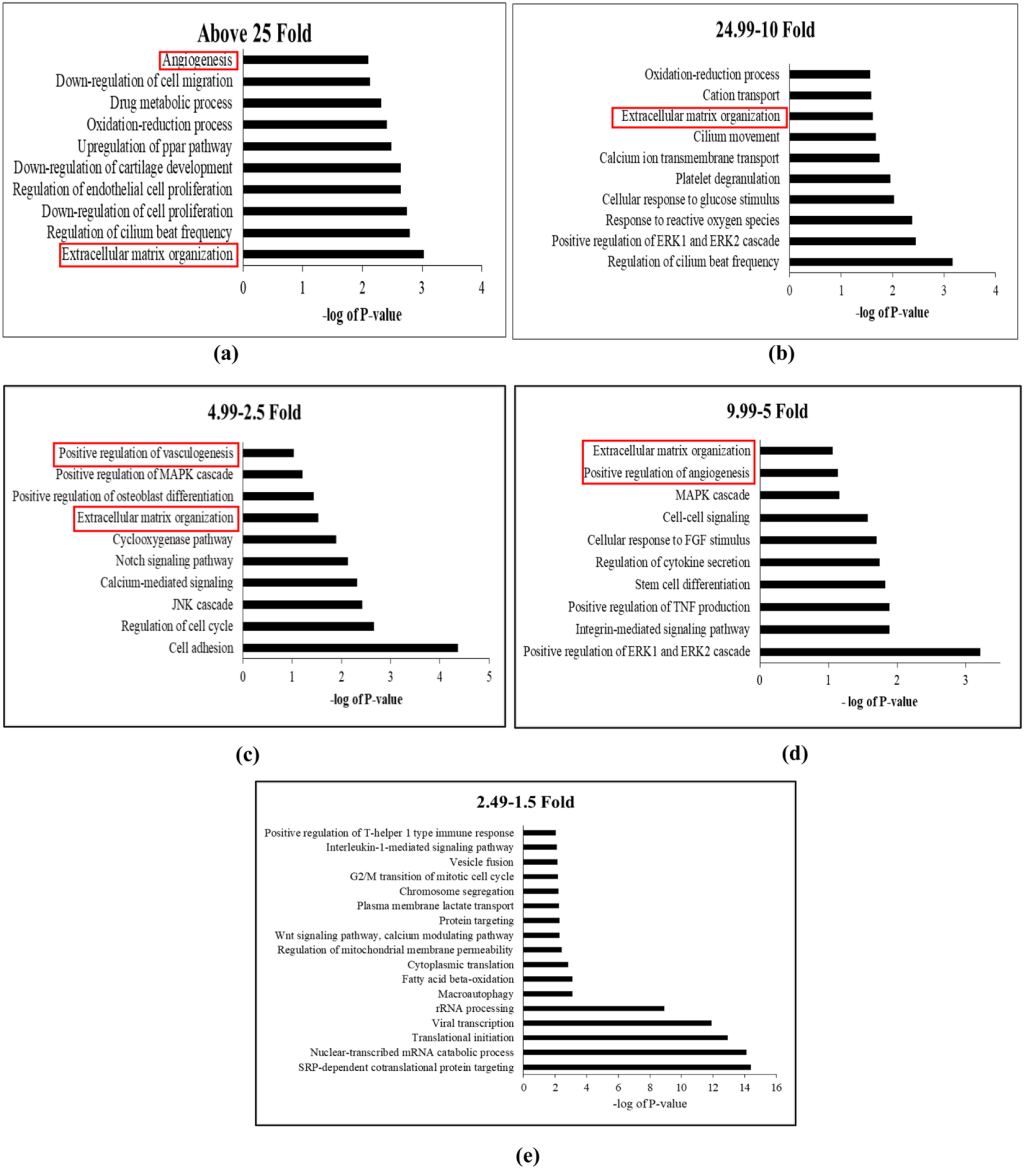
Gene Ontology Enrichment Analysis performed using David Bioinformatics Resources on the upregulated gene groups classified based on their fold changes (FC). (**a**) Genes above Fold change 25, (**b**) between 24.99–10 FC, (**c**) between 4.99–2.5 FC, (**d**) between 9.99–5 FC, (**e**) between 2.49–1.5 FC.

**Figure 3:**
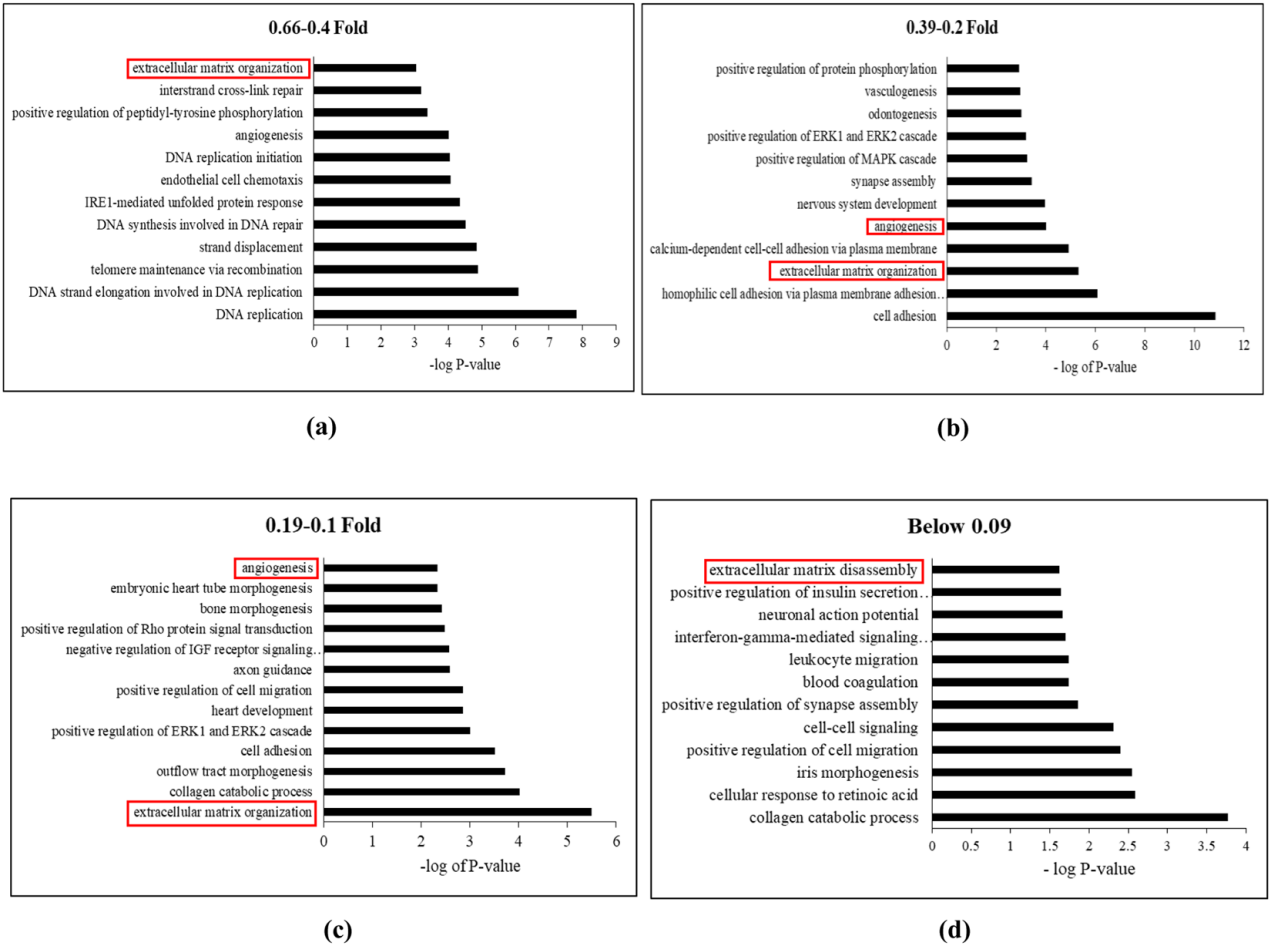
Gene Ontology Enrichment Analysis performed using David Bioinformatics Resources on the downregulated gene groups classified based on their fold changes (FC). (**a**) Genes between 0.66–0.4 FC, (**b**) between 0.39–0.2 FC, (**c**) between 0.19–0.1 FC, (**d**) below 0.09 FC.

To better understand the overall changes induced in ASCs following osteogenic differentiation, total gene changes were evaluated through non-biased means. Amongst all the gene changes, there are ninety three genes above 25 FC, two hundred and sixteen genes between 24.99–10 FC, five hundred and twenty eight genes between 9.99–5 FC, one thousand six hundred and four genes between 4.99–2.5 FC, and three thousand seventy three genes between 2.49–1.5 FC, and for the downregulated, there are one thousand four hundred and twenty two between 0.66–0.4 FC, seven hundred and three between 0.39–0.2 FC, two hundred and sixty six between 0.19–0.1 FC, and one hundred and seventy one below 0.09 FC (Fig. 1). Each cohort group mentioned above was separately subjected to functional annotation analysis using DAVID Bioinformatics Resources^25,27^ to find significantly enriched genes and associated pathways. The non-biased pathway analysis of upregulated gene groups revealed that there was a heavy association with pathways modulating extracellular matrix organization, angiogenesis, cell adhesion regulation of ERK1 and ERK2 cascade, MAPK cascade and regulation of endothelial cell proliferation (Fig. 2a–d). While the gene ontology analysis of downregulated genes below also suggested the enrichment of extracellular matrix organization and disassembly, angiogenesis, collagen catabolic process, MAPK cascade, ERK1 and ERK2 cascade, and calcium dependent cell-cell adhesion (Fig. 3a–d).

### Matrisome gene expression

**Figure 4:**
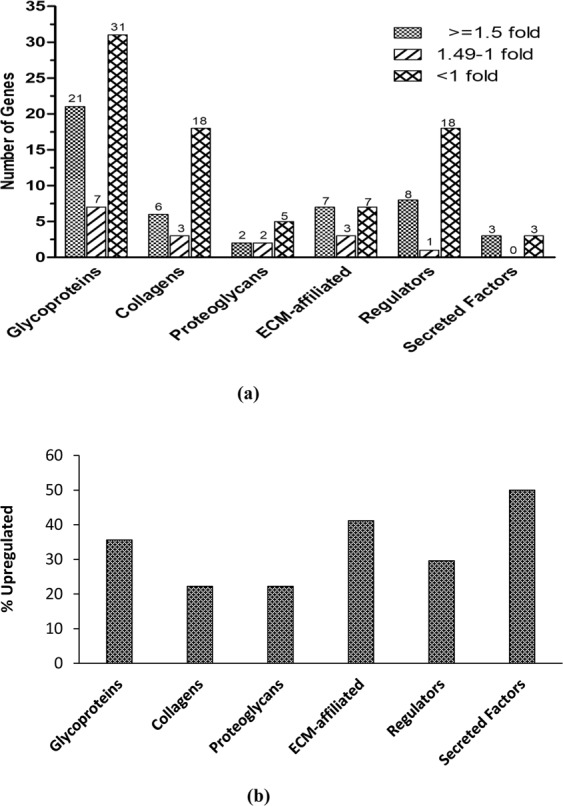
Gene expression of matrisome proteins; (**a**) (Top): Number of genes expressing in each sub-class of matrisome proteins. (**b**) (Bottom): Percentage of upregulated matrisome genes in each sub-class.

**Figure 5:**
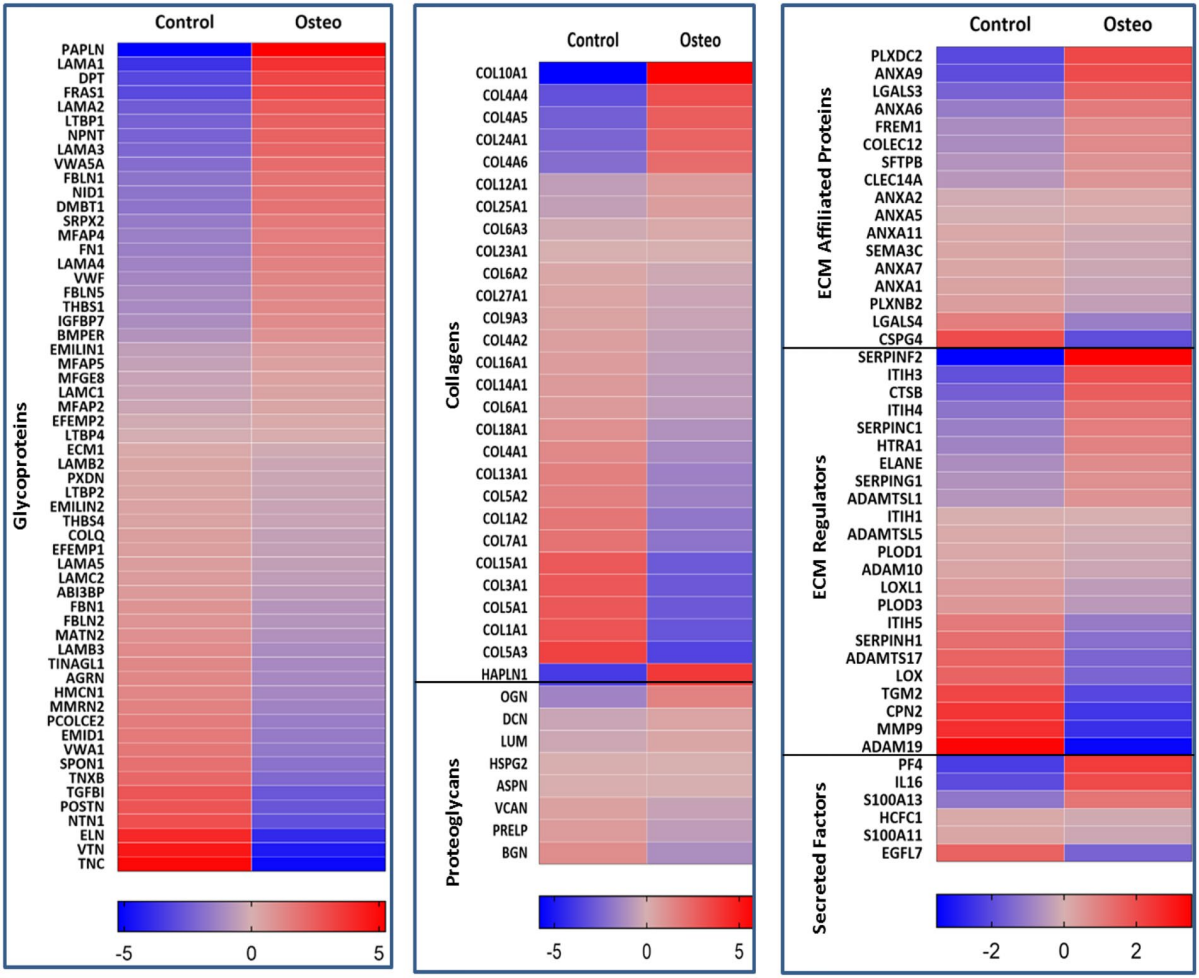
Gene expression of glycoproteins, collagens, proteoglycans, ECM affiliated ECM regulators, and secreted factors represented by heat maps. The log2 fold change values were used to generate heat maps.

The matrisome is defined as the set of all the proteins that comprise in the ECM; they are sub classified into glycoproteins, collagens, proteoglycans, ECM-affiliated, ECM-regulators, and secreted factors^7,28^. The list of genes encoding matrisome proteins was obtained from matrisome database (http://matrisomeproject.mit.edu/other-resources/human-matrisome/). We found that 35% of glycoproteins, 22% of collagens and proteoglycans, 41% of ECM-affiliated, 29% of regulators, and 50% of secreted factors were upregulated during osteogenesis in each matrisome sub-class (Fig. 4). It should also be noted that total number of genes in secreted factors and ECM-affiliated categories are low which resulted in high percentages than glycoproteins. The expression of matrisome genes in each subclass during osteogenesis was evaluated (Fig. 5, and Sup Tables S3–S8).

### Expression of ECM remodeling enzymes and Integrins

**Figure 6:**
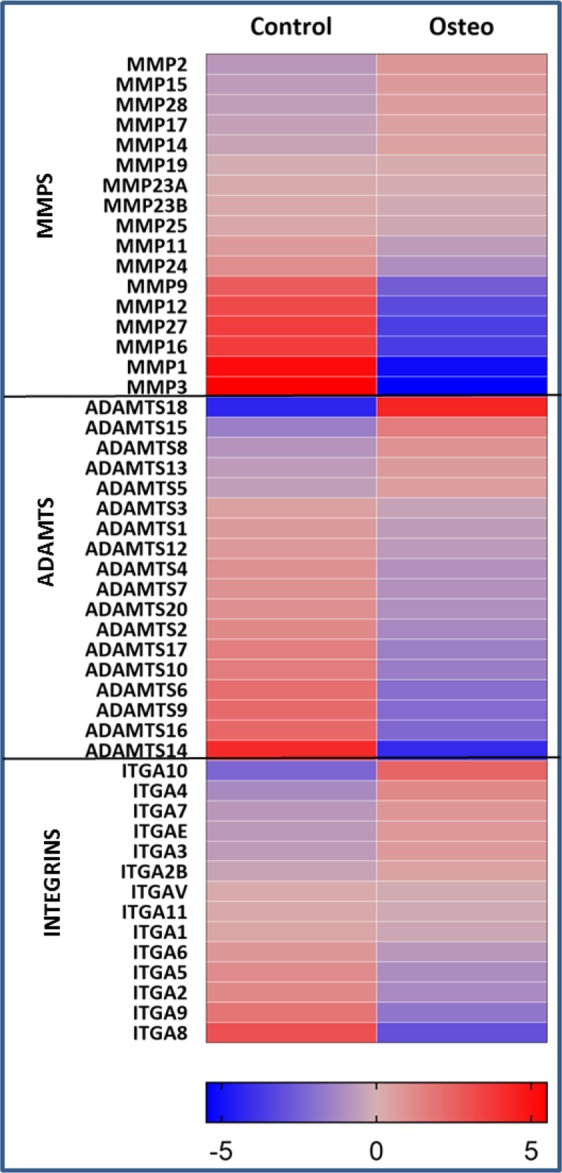
Gene expression changes of MMPs, ADAMTSs, and Integrins between osteogenic and undifferentiated ASCs as determined by RNA-Seq. The log2 fold change values were used to generate heat maps.

MMPs and ADAMTSs are the essential enzymes that participate in matrix remodeling. The list of MMPs and ADAMTSs genes was obtained from (https://www.genenames.org) and their expression was evaluated. There was downregulation of MMPs and ADAMTSs in the osteogenic induced ASCs relative to the undifferentiated ASCs (Fig. 6 and Sup Tables S9–S10). The MMPs with increased expression are MMP2, MMP15, and MMP28; while ADAMTSs 18, 15, 8, and 13 increased in expression with the osteogenic induction of ASCs (Fig. 6 and Sup Tables S9–S10). Integrins are heterodimer transmembrane molecules that are formed by combination of α and β chains and act as molecular bridges between cytoplasm and ECM. So far 24 α subunits and 9 β subunits have been identified in mammals which form different heterodimeric combinations to bind to various ECM proteins^29,30^. We found that during osteogenesis in ASCs, the alpha integrins ITAG 10, 4, 7, E, and 3 and beta integrins ITGB 2, 8, L1, and 4 shown increased expression (Fig. 6 and Sup Tables S11–S12).

### Osteogenic and Angiogenic genes expression

**Figure 7:**
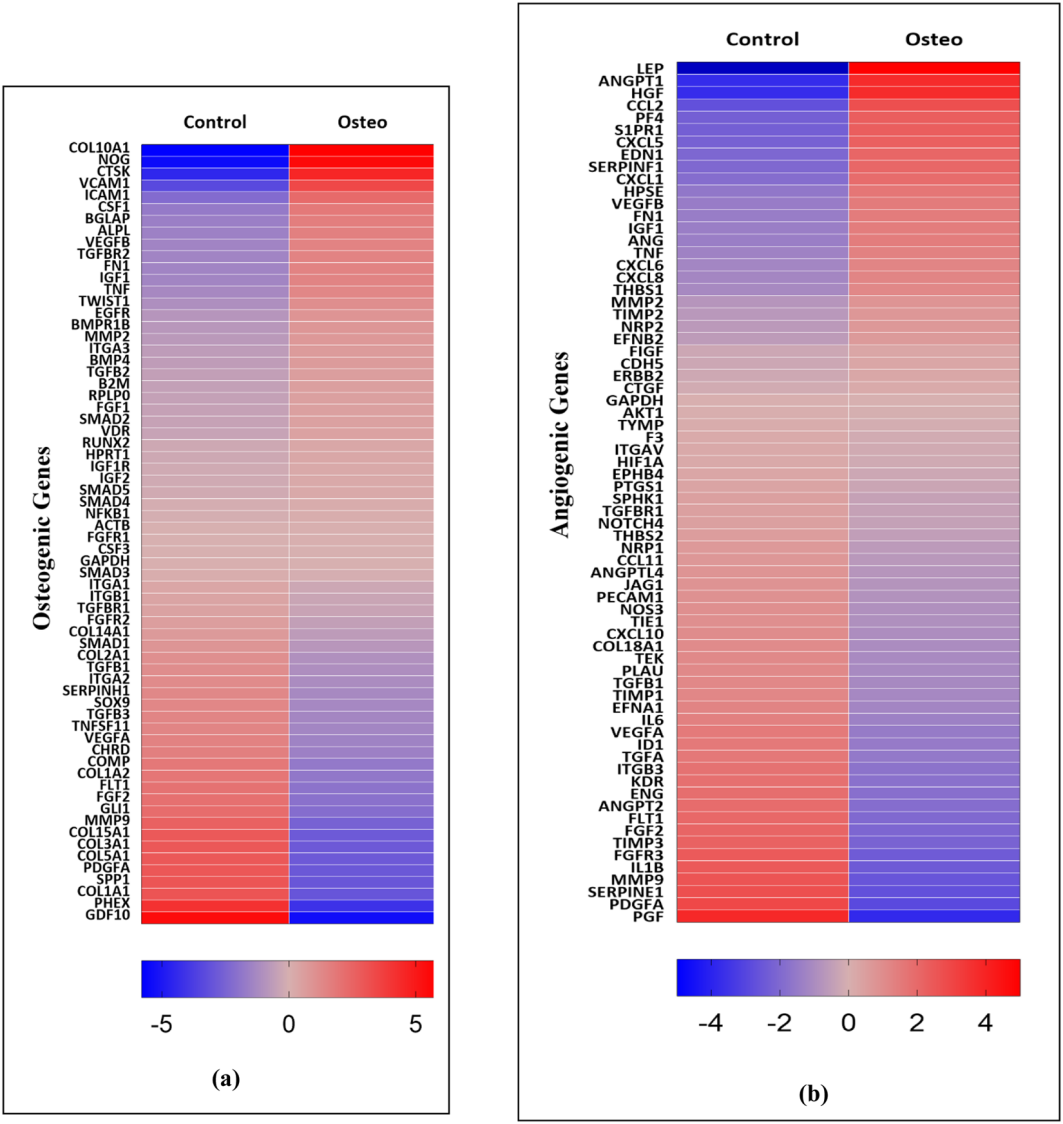
(**a**) Expression of osteogenic genes in ASCs undergoing osteogenesis after 21 days by RNA-Seq; (**b**) Expression of angiogenic genes in ASCs undergoing osteogenesis after 21 days by RNA-Seq.

The expression of the osteogenic and angiogenic genes are represented in the heatmaps (Fig. 7a,b) and in the Sup Tables (S2, S13). It was found that several osteogenic genes such as COL10A1, NOG, BGLAP, ALPL and angiogenic genes such as LEP, ANGPT1, HGF, and several CXC cytokines are upregulated during the osteogenesis of ASCs.

### Expression of regulatory genes in WNT, TGF-β, JNK, HedgeHog and ERK1/2 pathways

**Figure 8:**
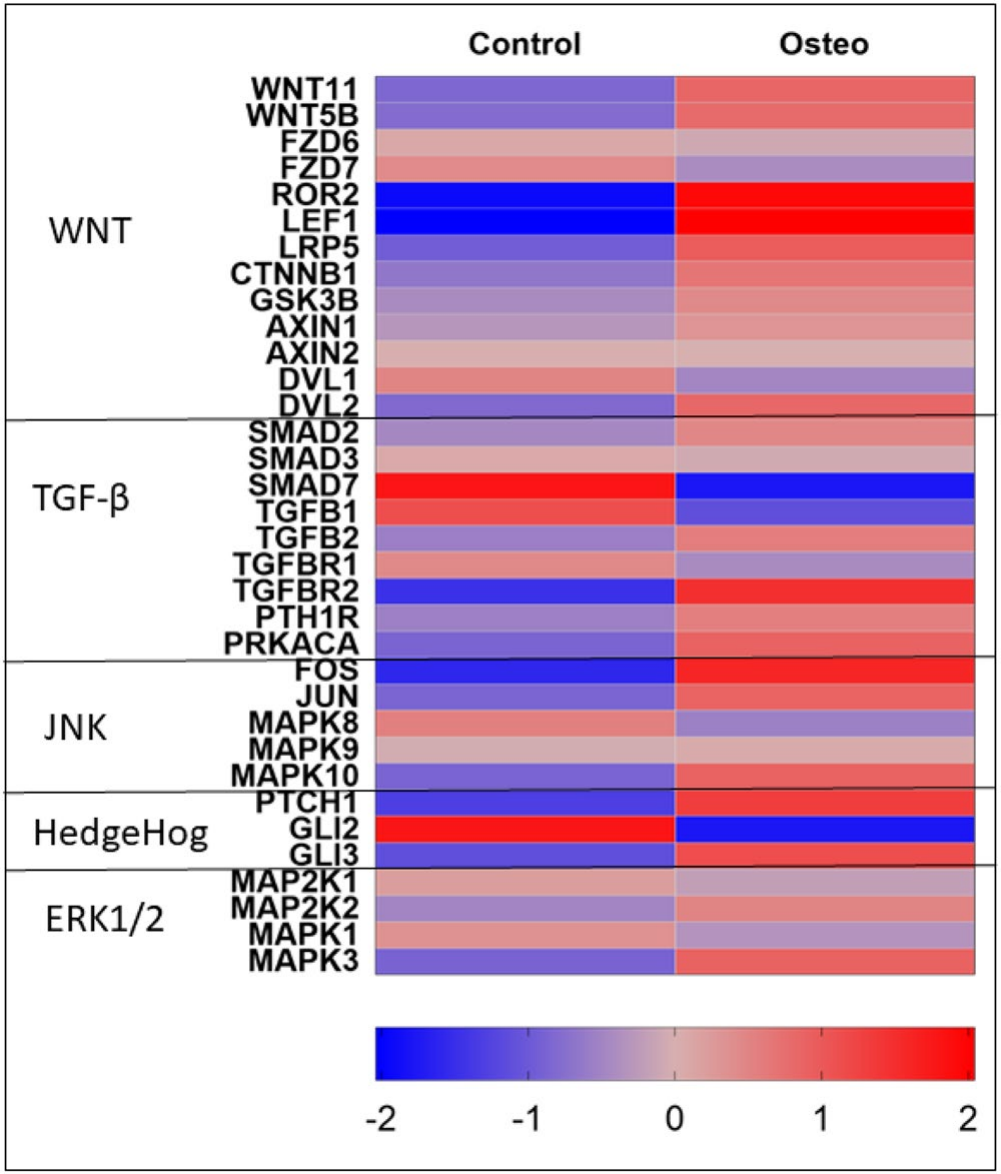
The expression of major regulators of WNT signaling pathway, TGF-β signaling, HedgeHog signaling, and ERK1/2 pathways are shown.

The expression of major regulatory genes involved in the WNT, TGF-β, JNK, HedgeHog and ERK1/2 pathways are shown in the Fig. 8 and Table 1. Major regulatory genes of these pathways such as CTNNB1, TGBR2, JUN, FOS, GLI3, and MAPK3 are upregulated indicating the regulation of osteogenesis through interplay between these pathways (Fig. 8 and Table 1).

**Table 1:** Expression of regulatory genes in WNT, TGF-β, JNK, HedgeHog and ERK1/2 pathways.

Gene	Control Count (CC)	Control FPKM	Osteo Count (OC)	Osteo FPKM	Fold Change (OC/CC)
WNT11	54.00	0.74	97.62	1.31	1.81
WNT5B	1791.93	25.60	3114.00	45.28	1.74
FZD6	1684.02	12.67	1581.63	12.35	0.94
FZD7	985.96	6.43	736.56	4.76	0.75
ROR2	983.81	6.29	3785.68	28.58	3.85
LEF1	9.00	0.29	37.00	0.35	4.11
LRP5	1447.00	7.45	2823.46	14.13	1.95
WNT9A	50.03	0.32	5.00	0.03	0.10
SFRP2	19264.29	247.69	2656.32	33.81	0.14
CTNNB1	4602.24	45.58	7320.38	78.25	1.59
GSK3B	1124.98	8.46	1519.08	10.62	1.35
AXIN1	580.00	4.15	717.62	5.39	1.24
AXIN2	222.85	2.71	222.74	2.18	1.00
DVL1	2227.00	22.56	1581.09	15.28	0.71
DVL2	1759.84	21.04	3116.55	38.01	1.77
SMAD2	2143.02	28.33	2968.44	43.56	1.39
SMAD3	5425.58	39.72	5155.08	37.06	0.95
SMAD7	2198.90	24.34	633.34	8.12	0.29
TGFB1	4702.29	71.86	2142.55	37.60	0.46
TGFB2	80.00	0.83	118.60	0.58	1.48
TGFBR1	4759.46	41.74	3530.18	34.88	0.74
TGFBR2	14775.10	79.97	41096.61	220.77	2.78
PTH1R	79.00	2.06	117.00	2.06	1.48
PRKACA	429.83	4.60	794.39	9.58	1.85
FOS	107.00	1.50	323.40	4.24	3.02
JUN	1333.68	9.51	2454.04	17.36	1.84
MAPK8	566.99	17.71	385.81	11.92	0.68
MAPK9	1644.04	14.31	1702.58	15.01	1.04
MAPK10	29.00	0.40	53.71	0.65	1.85
PTCH1	135.29	1.25	337.14	3.01	2.49
GLI2	764.97	13.16	220.89	4.16	0.29
GLI3	908.90	6.03	1992.76	13.80	2.19
MAP2K1	2835.54	28.45	2462.08	25.17	0.87
MAP2K2	4909.45	106.62	6965.68	146.39	1.42
MAPK1	10938.19	127.56	8654.65	100.43	0.79
MAPK3	925.06	15.90	1719.48	30.11	1.86

### RNA-Seq validation of differentially expressed genes

**Figure 9:**
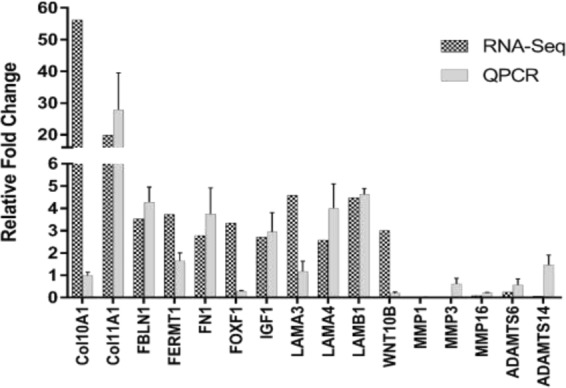
RNA-Seq validation of upregulated and downregulated genes by QPCR. Comparison of the expression changes of 16 genes COL10A1, COL11A1, FBLN, FERMT1, FN1, FOXF1, LAMA3, LAMA4, LAMB1, IGF1, WNT10B, MMP1, MMP3, MMP16, ADAMTS6, and ADAMTS14 as determined by RNA-Seq and QPCR.

**Figure 10:**
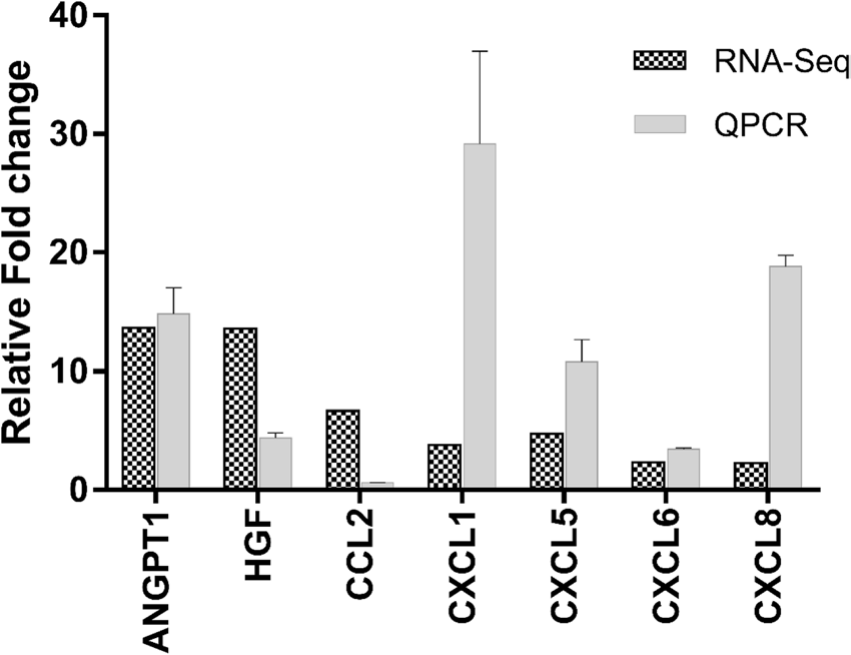
Expression of pro-angiogenic cytokines during osteogenesis. The upregulation of pro-angiogenic cytokines determined using RNA-Seq is also observed by QPCR with the exception of CCL2 gene.

To confirm the RNA-Seq data we have selected the following 16 upregulated genes (LAMA4, FN1, LAMA3, COL10A1, COL11A1, IGF1, FOXF1, FBLN1, WNT10B, and LAMB1) and 5 downregulated genes (MMP1, MMP3, MMP16, ADAMTS6, and ADAMTS14) that are known to have prominent role in the extracellular matrix organization, and cell adhesion pathways. Expression was measured by QPCR and normalized to GAPDH. QPCR evaluation indicates the expression changes of LAMA4, IGF1, FBLN1, LAMB1, FN1, COL11A1, MMP1, MMP3, MMP16 and ADAMTS6 were found to be correlated with the expression changes of these genes revealed by RNA-Seq (Fig. 9 and Table 2). Note that ADAMTS14 was shown to be neither downregulated nor upregulated in the QPCR data. Similar observations can be made for COL10A1, FOXF1, LAMA3 and WNT10B where the QPCR data is not in conformity with the RNA-Seq data. The upregulation of pro-angiogenic cytokines (ANGPT1, HGF and CXC) as well as the ELR+ chemokines (CXCL1, CXCL5, CXCL6, and CXCL8) was also evaluated by QPCR (Fig. 10). The data suggested an increased expression for these cyto- and chemo-kines with the exception of CCL2 gene during osteogenesis of ASCs (Fig. 10).

**Table 2:** Comparison between RNA-Seq and QPCR.

RNA-Seq				QPCR
Gene	Control Counts	Osteo Counts	Fold Change	Fold Change
Col10A1	5.00	281.00	56.20	0.98 ± 0.14
Col11A1	545.58	10800.60	19.80	27.83 ± 11.71
FBLN1	37245.64	130866.10	3.51	4.27 ± 0.67
FERMT1	18.00	67.00	3.72	1.64 ± 0.35
FN1	1222356.00	3391541.00	2.77	3.74 ± 1.17
FOXF1	1.00	3.34	3.34	0.28 ± 0.03
IGF1	50.92	137.92	2.71	2.94 ± 0.86
LAMA3	93.00	425.97	4.58	1.17 ± 0.45
LAMA4	14447.68	37124.57	2.57	4.00 ± 1.09
LAMB1	8165.41	36493.15	4.47	4.62 ± 0.26
WNT10B	1.00	3.00	3.00	0.19 ± 0.05
MMP1	2327.16	64.99	0.02	0.03 ± 0.01
MMP3	2299.93	51	0.02	0.61 ± 0.26
MMP16	599.47	48.02	0.08	0.22 ± 0.02
ADAMTS6	764.97	184	0.24	0.56 ± 0.28
ADAMTS14	833.36	45.01	0.05	1.46 ± 0.45
ANGPT1	120	1639.72	13.66	14.83 ± 2.17
HGF	68.79	935.39	13.59	4.38 ± 0.44
CCL2	1101.39	7375.72	6.69	0.61 ± 0.01
CXCL1	32	121	3.78	29.16 ± 7.81
CXCL5	79	376.09	4.76	10.83 ± 1.84
CXCL6	59	136.87	2.31	3.46 ± 0.10
CXCL8	38	87	2.28	18.83 ± 0.90

## Discussion

The role of ECM was once presumed mainly to be static and limited to providing structural support as a matrix. Recent studies have shown the regulatory role of ECM^31,32^ and secretome in the differentiation process^33^. Thus, the development of bone and vasculature and their integration with each other requires robust cell to cell communication between osteoblasts through ECM and secretome. The comprehensive transcriptomic characterization of ECM from ASCs, BMSCs and other MSCs has been reported in literature^34^. However, to our knowledge this is the first study to characterize and correlate the ECM transcriptome and the secretome of ASCs undergoing osteogenesis. GO analysis in biological process category revealed the enrichment of ECM and angiogenesis pathways with both upregulated and downregulated genes in every FC cohort group except 2.49–1.5 FC (Figs 2, 3). This output from GO analysis suggests that the potential regulatory role of ECM synthesis and secretome linked to osteogenic differentiation in ASCs. Therefore, we focused on the expression of the ECM genes which make the core matrisome proteins (collagens, glycoproteins and proteoglycans), matrisome associated proteins (ECM-affiliated proteins, ECM-regulators, and secreted factors), ECM remodeling enzymes and secretomic genes. The expression levels of all these matrisome encoding genes under each sub-class in the ASCs undergoing osteogenesis was evaluated (Figs 4–6, Sup Tables S3–S8, S9–S12).

Collagens are the important components of ECM and also play a crucial role in osteogenesis. Among the upregulated collagens, the expression of COL10A1, COL4A4, COL4A5, COL4A6, COL12A1 and COL24A1 was found to be increased (Fig. 5 and Sup Table S4). COL12A1 and COL24A1 are previously reported to have a role in the regulation of osteogenesis. For example, COL12A1 is crucial for the terminal osteogenic differentiation and also helps in cell-cell communications by regulating gap junction protein, GJA1^35^. It was found the null mice with COL12A1 deletion showed altered bone development with reduced bone mass^35^. COL24A1 overexpression enhances osteogenesis while silencing COL24A1 reduces osteogenic potential. COL24A1 interacts with the integrin β3 chain to activate TGF-β/SMADS signaling pathway and thus regulates osteogenesis^36^. COL10A1 is a key marker in the chondrogenesis as its induction will lead to the chondrogenic differentiation in MSCs^37,38^.

Glycoproteins play important roles in the ECM assembly, cell signaling and binding to growth factors^8^. Laminins and fibulins are well known categories of glycoproteins. Laminins are the heterotrimeric glycoproteins, with 3 different chains α, β, and γ, that are found primarily in the basement membrane and mediate cell adhesion, proliferation, migration, differentiation and angiogenesis^39^. In laminins, we have the upregulation of LAMA1, LAMA2, LAMA3, and LAMA4 (Fig. 5, Sup Table S3). The pro-angiogenic properties of Laminin alfa 1(LAMA1) chain have been well documented. The peptide with amino acid sequence SIKVAV derived from LAMA 1 when injected into nude mice along with melanoma cells promoted tumor growth by enhancing the angiogenesis of the tumor^40^. Similarly, A13 and AG73 peptides from LAMA1 were found to be robust angiogenic inducers^41,42^. Furthermore, C57BL/6J mice with a missense mutation in the LAMA1 gene develops abnormal retinal vasculature leading to impaired inner limiting membrane formation^43^. On the other hand, transfecting LAMA1 chain in HT29 colonic cancer cells increased angiogenesis by recruiting fibroblasts that led to the significant growth in tumor^28^.

LAMA4 subunit expression restricted to certain tissues and is mainly found in vascular endothelial basement membranes of brain, muscle, and bone marrow. Antibodies against the G1/G2 domain of LAMA4 subunit impede the branching morphogenesis of microvascular endothelial cells^44^. LAMA4 deficient mice develops impaired microvessel organization with poor structural integrity leading to hemorrhage in subcutaneous tissues^45^. More importantly, the G domain of LAMA4 chain has shown to exhibit high affinity to bind to integrins, α3β1 and αVβ3, on endothelial cells which ultimately leads to angiogenesis^46^. LAMA2 is majorly found in the basement membrane surrounding skeletal muscle and mutation in this gene can cause poor muscle development leading to muscular dystrophy^47^. Laminin 332, similar to other laminins is found in basement membranes, aids in cell migration and invasion. The interactions between LAMA3 subunit of laminin 332 with α6β4 integrin and syndecan 1 leads to the activation of protein kinase C (PKC) pathway which causes tumor cell migration and invasion^48^.

The two fibulins, FBLN1 and FBLN5, are found to be upregulated during osteogenesis (Fig. 5, Sup Table S3). FBLN1 enhances the osteogenesis (both endochondral and intramembranous) by positively regulating of BMP2 and Osterix^49^. Lack of FBLN1 expression in the perinatal mice resulted in the improper skull development and premature death^50^. In FBLN5 deficient mice pre-maxillary bone defects are observed and FBLN5 is crucial for the MSCs proliferation within the facial suture^51^. Other notable glycoproteins upregulated that are known to regulate osteogenesis and angiogenesis are Fibronectin FN1, BMPER, VWF, THBS1, FRAS1, and NID1 (Fig. 5, Sup Table S3).

Proteoglycans are mainly found in the ECM of connective tissues and facilitate hydrodynamic swelling of the tissue to sustain the external compressional forces^52^. HAPLN1 and Osteoglycin (OGN) are two proteoglycans that are increased in expression due to osteogenic induction in ASCs (Fig. 5, Sup Table S5). In MC3T3-E1 cells, overexpression of OGN reduced the expression of RUNX2 and Osterix while significantly upregulated ALPL, type I collagen (Col1), and osteocalcin (OCN), β-catenin and calcium mineralization^53^.

The ECM remodeling enzymes (MMPs and ADAMTSs) are known to degrade ECM as a part of ECM repair and remodel process. The MMPs 2, 5, 28 and the ADAMTSs 18, 15, 8, 13 are overexpressed in the ASCs undergoing osteogenesis (Fig. 6, Tables S9 and S10). MMP2 is known to play key role in the wound healing process by degrading type IV collagen^54^ and is also found to be significantly expressed by osteoarthritic cartilage^55^. MMP28 is also known to be involved in wound healing and repair process in the injured keratinocytes and testis^56^. ADAMTS18 is a bone mass candidate gene where the loss of expression is directly correlated to skeletal fractures^57^. ADAMTS8 and ADAMTs15 are shown to be the clinical markers of breast cancers^58^ whereas ADAMTS13 is known to regulate blood clotting through mediating the von Willebrand factor (VWF)^59^.

The overall fate of angiogenesis is dependent on the balance between angiogenic and angiostatic factors. CXC chemokines contain two conserved cysteine (C) residues towards the amino terminus and are separated by any other amino acid represented as X. The CXC chemokines with amino acids, glutamic acid-leucine-arginine (termed as ELR motif) towards the amino terminal side of the first cysteine residue are ELR positive CXC chemokine which are regarded as proangiogenic while the CXC chemokines without ELR motif are angiostatic^60,61^. Examples of ELR+ CXC chemokines are CXCL1, CXCL2, CXCL3, CXCL5, CXCL6, CXCL7, and CXCL8. We found increased expression of ELR+ chemokines especially CXCL1, CXCL5, CXCL6 and CXCL8 during osteogenesis (Fig. 7b and Sup Table S13). It was demonstrated that CXCL1 secreted by human endothelial cells increases rate of angiogenesis by autocrine response and effects surrounding epithelial cells by paracrine signaling while blocking CXCL1 resulted in decreased ability to induce angiogenesis^62^. Furthermore, CXCL1 results in the expression of EGF, another angiogenic inducer, through ERK1/2 pathways mediation^62^. Consistent with this, we found the upregulation ERK1/2 pathways indicating CXCL1 mediated autocrine signaling in osteogenic induced ASCs (Fig. 2b,d). CXCL8 is another angiogenic chemokine whose expression was found to be increased in osteogenesis. Similar to CXCL1, CXCL8’s expression correlates to the angiogenic development in human pancreatic, ovarian and non-small lung cancer (NSLC)^63^. CXCL5, along with CXCL8, also plays a crucial role in mediating angiogenesis in NSLC whose expression levels correlates with angiogenic potential^64^. In MSCs, overexpression of CXCL6 enhances the upregulation of pro-angiogenic genes such as VEGF-A, IGF-1, HGF and CXCL8^65^. The secretome from cardiac progenitor cells containing CXCL6, CXCL1, and CXCL8 promoted angiogenic activity of HUVEC cells^66^. QPCR studies performed to confirm the upregulation of ELR+ chemokines (CXCL1, CXCL5, CXCL6, and CXCL8) and other pro-angiogenic cytokines (ANGPT1, HGF, and CCL2) suggested the increased expression of these genes (Fig. 10 and Table 2). Contrary to the upregulation of CCL2 gene as indicated by RNA-Seq, we found the downregulation of CCL2 by QPCR while all other pro-angiogenic genes studied by QPCR showed increased expression as indicated by RNA-Seq (Fig. 10 and Table 2). The upregulation of these ELR+ chemokines during osteogenesis suggests the possible paracrine effect to induce angiogenesis of the endothelial progenitor cells in vicinity.

The expression of major regulators of WNT signaling pathway, TGF-β signaling, HedgeHog, and ERK pathways are shown (Fig. 8). WNT signaling has been shown to upregulate osteogenesis through both β-catenin dependent and β-catenin independent pathways^67–69^. The binding of WNT ligands to the frizzled receptors (Frz) facilitates a complex formation that involves a transmembrane receptor low-density lipoprotein receptor-related protein 5 (LRP5) with a disheveled proteins (DVL1, DVL2)^70^. The disheveled proteins inhibit the complex (Axin, GSK3β, APC) that degrades β-catenin (CTBBN1) which eventually translocates to the nucleus, hetero-dimerizes with lymphoid enhancer-binding factor/T cell factor (LEF/TCF) and regulates transcriptional process. The expression of CTBBN1, LRP5, LEF1, and DVL2 are found to be increased during osteogenesis of ASCs indicating that the β-catenin dependent canonical WNT signaling pathway is likely regulating osteogenesis (Fig. 8, Table 1). TGF-β signaling is also in regulating osteogenesis through TGF-β receptors and SMADs^3^. The binding of ligands to TGF-β receptors (TGFBR1 and TGFBR2) elicits the SMAD proteins especially SMAD2, SMAD3, and SMAD4 to form a complex that translocates to nucleus to regulate gene activity whereas SMAD7 disintegrates the SMAD2/3 and SMAD4 complex preventing it from entering the nucleus^3^. The expression of TGFBR2 is increased and SMAD7 (inhibitor of SMAD2/3 and SMAD4 complex) expression decreased while the expression of SMAD2/3 and SMAD4 remained unchanged in our data suggesting the possibility of TGF-β regulation of osteogenesis in ASCs (Fig. 8, Table 1). HedgeHog pathway is also known to influence osteogenic pathway through the transcription factor Gli3^67^. The expression of Gli3 was found to be upregulated suggesting the likelihood of hedgehog pathway’s involvement (Fig. 8, Table 1).

The AP-1 complex proteins mainly c-JUN (JUN) and c-FOS and the MAPK10 (JNK3 enzyme) are found to be upregulated during osteogenesis indicating that JNK pathway is also involved in osteogenic regulation. The expression of MAPK3, a major enzyme of ERK1/2 pathway, was found to be increased suggesting that ERK1/2 pathway involvement in osteogenesis (Fig. 8, Table 1). The upregulation of both MAPK signaling pathways (JNK and ERK1/2) was also indicated by GO functional analysis (Fig. 2b–d) as these two pathways were significantly enriched. The enrichment MAPK signaling pathways and AP1 transcription factor (JUN) has also been demonstrated in a recent study by Martin *et al*., where ASCs were treated with serum from heterotopic ossification patients in a ectopic bone formation model^71^. Overall, this data suggests that the interplay and crosstalk between WNT, TGF-β, JNK, HedgeHog and ERK1/2 pathways regulates osteogenesis in ASCs through the ECM and pro-angiogenic secretome release that could function as paracrine signaling facilitating the vascularization of bone tissue.

### Comparative analysis between ASCs and BMSCs secretome during osteogenesis

**Figure 11:**
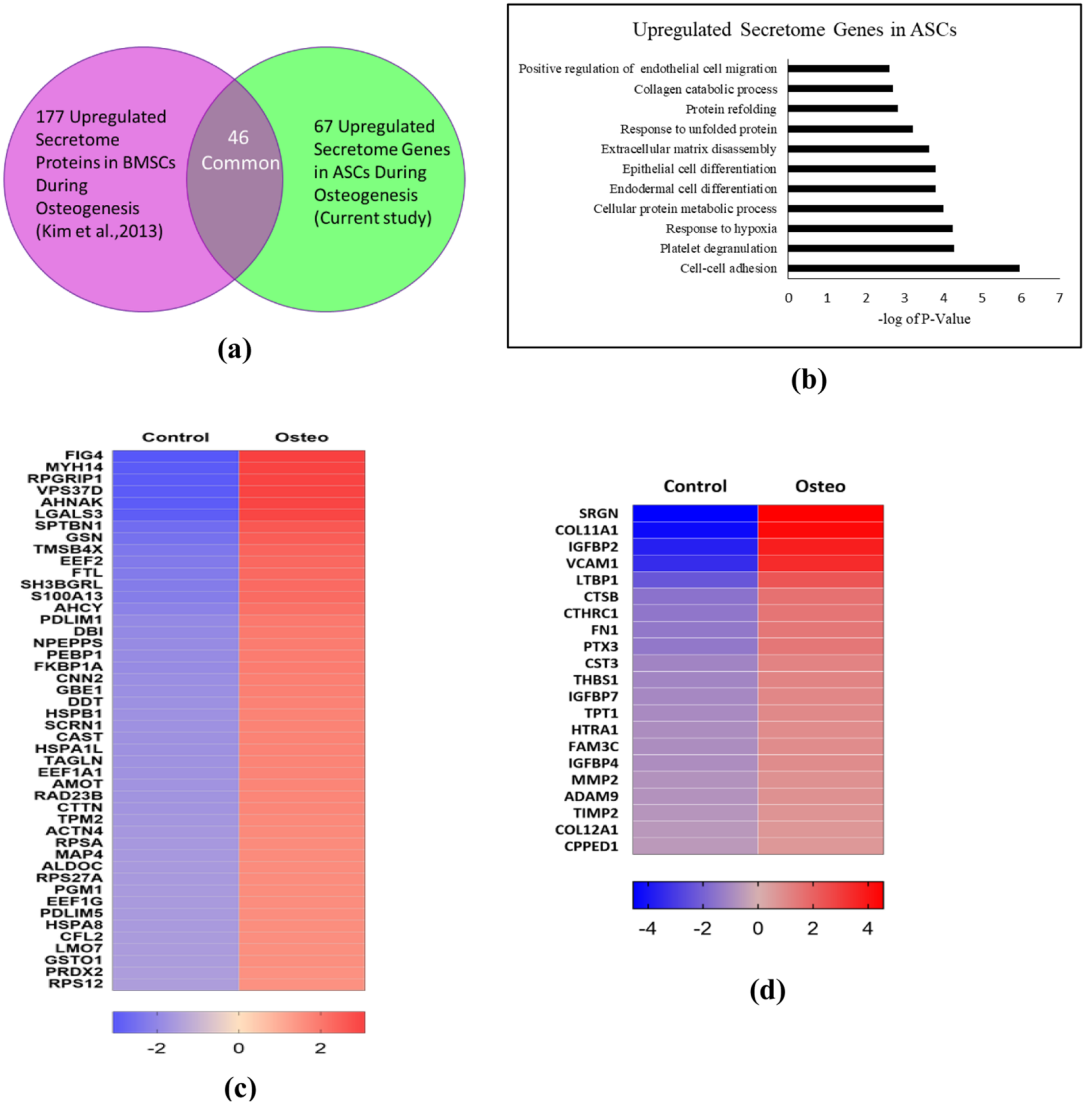
The expression of secretome genes exclusive for ASCs during osteogenesis. (**a**) Venn diagram representing the comparison of secretome genes between BMSCs and ASCs during osteogenesis. The 177 upregulated secretome proteins includes 46 common genes and 131 genes exclusively found in BMSCs. Similarly, the 67 upregulated proteins includes 46 common genes and 21 genes exclusive to ASCs. (**b**) Gene ontology functional analysis of upregulated secretome genes in ASCs under biological process category. (**c**) Representation of the 46 common upregulated genes between ASCs and BMSCs. (**d**) Twenty one genes were found to be exclusively upregulated in ASCs that were downregulated in BMSCs; Representation of 21 exclusively upregulated secretome genes in ASCs during osteogenesis.

The secretome of BMSCs undergoing osteogenesis was analyzed by Kim *et al*.^72^ using LC-MS/MS technique. Kim *et al*., identified 315 secretome proteins out of which 177 were upregulated during osteogenesis of BMSCs (Fig. 11a). We have examined the genes coding for those 315 proteins identified by Kim *et al*.^72^ in our ASC data. In the ASCs, during the osteogenic differentiation, 67 secretome genes were found to be upregulated of which 46 of them were found to be in common between ASCs and BMSCs (Fig. 11a,c and Table 3). There are 21 genes (the difference between 67 and 46) which are upregulated exclusively in ASCs (Fig. 11d and Table 4). Gene ontology (GO) analysis performed on each of these categories suggest the enriched pathways as cell-cell adhesion, collagen catabolic process, endothelial cell migration, and ECM regulation with all the upregulated ASC secretome genes (Fig. 11b). While the GO analysis with the genes exclusively expressed in ASCs revealed the enrichment of insulin-like growth factor (IGF) receptor signaling pathway, ECM regulation, response to hypoxia, collagen catabolic process, cell adhesion pathways (Sup Fig. S3). Although up- and down-regulated pathways/proteins/genes during BMSCs osteogenesis are reported in the literature^33,67,70,72^, the raw RNA-Seq data that will be enable us to perform a comparative analysis isn’t easily accessible. Further studies are clearly required to better our understanding of the differences and the commonalities in the differentiation pathways of BMSCs and ASCs.

**Table 3:** Upregulated Common Secretome Genes between ASCs and BMSCs.

Gene	Control Count (CC)	Control FPKM	Osteo Count (OC)	Osteo FPKM	Fold Change (OC/CC)
FIG4	544.03	4.84	1675.97	14.38	3.08
MYH14	1	0.01	3.01	0.01	3.01
RPGRIP1	1	0.01	3	0.04	3
VPS37D	16	0.32	48	0.78	3
AHNAK	12132.45	79.99	36115.7	135.96	2.98
LGALS3	6197.81	214.73	18369.56	607.02	2.96
SPTBN1	5923.66	17.05	15472.51	44.27	2.61
GSN	13723.85	142.23	34481.69	342.32	2.51
TMSB4X	151621.9	7675.33	361105.8	17855.98	2.38
EEF2	15942.91	194.19	36593.72	417.97	2.3
FTL	201179.1	6708.51	459786.7	15057.5	2.29
SH3BGRL	1594	22.87	3604.26	55.1	2.26
S100A13	4311.37	215.58	9683.15	487.87	2.25
AHCY	2182.72	27.82	4623.48	56.1	2.12
PDLIM1	3293.45	64.87	6497.59	125.26	1.97
DBI	2215.56	110.27	4349.96	211.35	1.96
NPEPPS	2686.97	27.28	5157.74	51.27	1.92
PEBP1	5154.32	82.34	9880.23	156.3	1.92
FKBP1A	4932.81	131.67	9453.96	254.75	1.92
CNN2	9507.53	145.71	17678.32	287.58	1.86
GBE1	4573.82	40.56	8415.44	73.61	1.84
DDT	729.49	46.3	1312	95.21	1.8
HSPB1	7900.95	255.19	14131.4	441.36	1.79
SCRN1	7802.09	38.29	13949.53	72.82	1.79
CAST	3612.26	46.44	6441.29	88.46	1.78
HSPA1L	41.04	0.41	73.04	0.73	1.78
TAGLN	24600.35	882.13	43504.04	1507.41	1.77
EEF1A1	129725.6	1731.55	227632.6	3015.19	1.75
AMOT	34	0.21	59	0.53	1.74
RAD23B	984.41	6.73	1701.35	10.39	1.73
CTTN	6249.58	57.39	10716.97	100.68	1.71
TPM2	30125.95	625.52	50360.31	1004.06	1.67
ACTN4	2162.61	26.71	3551.22	37.81	1.64
RPSA	11390.87	274.38	18508.11	442.78	1.62
MAP4	17604.14	122.59	28564.79	214.31	1.62
ALDOC	269.37	4.23	432	6.57	1.6
RPS27A	12341.8	395.16	19762.15	614.94	1.6
PGM1	3813.51	40.26	6073.19	63.25	1.59
EEF1G	17832.26	314.76	28299.97	493.15	1.59
PDLIM5	4369.17	70.32	6930.35	111.21	1.59
HSPA8	24738.1	386.96	39034.45	625.1	1.58
CFL2	1823.38	42.15	2843.95	63.71	1.56
LMO7	5126.86	61.06	7980.4	105.15	1.56
GSTO1	4637.47	143.36	7178.85	214.55	1.55
PRDX2	3049.21	88.69	4619.64	133.47	1.52
RPS12	7138.81	361.07	10769.94	531.45	1.51

**Table 4:** Secretome Genes Exclusively Upregulated in ASCs.

Gene Name	Control	Control	Osteo	Osteo FPKM	Fold change (OC/CC)
	Count (CC)	FPKM	Count (OC)		
SRGN	158.00	3.62	3697.73	84.51	23.40
COL11A1	545.58	2.91	10800.60	46.77	19.80
IGFBP2	106.01	2.15	1428.76	32.55	13.48
VCAM1	75.00	0.62	801.89	6.58	10.69
LTBP1	2924.00	19.74	14915.07	108.48	5.10
CTSB	91894.27	1282.22	282894.50	3819.71	3.08
CTHRC1	5076.21	144.23	14407.83	446.09	2.84
FN1	1222356.00	4839.91	3391541.00	12484.05	2.77
PTX3	59930.91	810.25	164989.80	2207.37	2.75
CST3	6566.97	223.63	14576.58	489.93	2.22
THBS1	72760.73	351.28	159590.70	873.93	2.19
IGFBP7	10382.54	225.71	21559.07	454.89	2.08
TPT1	9248.75	250.27	18336.73	524.83	1.98
HTRA1	41243.08	696.12	78015.36	1305.63	1.89
FAM3C	2197.27	22.76	4153.87	43.09	1.89
IGFBP4	45780.88	540.30	85875.61	1003.59	1.88
MMP2	84805.54	1162.58	146515.20	1924.06	1.73
ADAM9	9804.65	70.22	16845.59	119.40	1.72
TIMP2	2247.62	15.61	3657.11	25.26	1.63
COL12A1	15090.88	57.83	23514.44	84.27	1.56
CPPED1	1545.70	13.49	2338.56	19.49	1.51

## Conclusion

In this study, we used RNA-Seq to determine the differential gene expression between undifferentiated ASCs and ASCs induced into osteogenesis on day 21. The differentially expressed genes were subjected to gene ontology functional analysis that revealed the enrichment of ECM and angiogenesis along with ERK1/2 and JNK pathways. The expression of matrisome genes (glycoproteins, collagens, and proteoglycans) and the expression of ECM remodeling enzymes (MMPs and ADAMTSs) and integrins with respect to osteogenesis in ASCs has been elucidated. The change in the expression patterns of pro-angiogenic genes between differentiated and undifferentiated ASCs into osteogenic lineage was evaluated and it indicates the secretion of several pro-angiogenic ELR+ chemokines and other angiogenic inducers suggesting the possible regulatory role of differentiating ASCs in the development of vasculature to accomplish functional bone formation. The depicted results suggest that the ECM and angiogenic gene expression varies with the induction of osteogenesis in ASCs. The expression of regulatory genes such as CTNNB1, TGBR2, JUN, FOS, GLI3, and MAPK3 involved in the WNT, TGF-β, JNK, HedgeHog and ERK1/2 pathways are found to be upregulated suggesting the regulation of osteogenesis through interplay between these pathways. Further validation of RNA-Seq data was performed by QPCR.

## Supplementary information

Supplementary Information

## Data Availability

The datasets generated during and/or analyzed during the current study are available from the corresponding author on reasonable request.

## References

[1] Soltanoff CS, Chen W, Yang S, Li Y-P (2009). Signaling networks that control the lineage commitment and differentiation of bone cells. Critical Reviews in Eukaryotic Gene Expression.

[2] Huang W, Yang S, Shao J, Li Y-P (2007). Signaling and transcriptional regulation in osteoblast commitment and differentiation. Frontiers in Bioscience.

[3] Chen G, Deng C, Li Y-P (2012). TGF-β and BMP signaling in osteoblast differentiation and bone formation. International Journal of Biological Sciences.

[4] Watt FM, Huck WT (2013). Role of the extracellular matrix in regulating stem cell fate. Nature Reviews Molecular Cell Biology.

[5] Fitzpatrick LE, McDevitt TC (2015). Cell-derived matrices for tissue engineering and regenerative medicine applications. Biomaterials Science.

[6] Manabe R-i (2008). Transcriptome-based systematic identification of extracellular matrix proteins. Proceedings of the National Academy of Sciences.

[7] Naba A (2011). The matrisome: In silico definition and *in vivo* characterization by proteomics of normal and tumor extracellular matrices. Molecular & Cellular Proteomics.

[8] Hynes RO, Naba A (2012). Overview of the matrisome—an inventory of extracellular matrix constituents and functions. Cold Spring Harbor Perspectives in. Biology.

[9] Engler AJ, Sen S, Sweeney HL, Discher DE (2006). Matrix elasticity directs stem cell lineage specification. Cell.

[10] Nam J, Johnson J, Lannutti JJ, Agarwal S (2011). Modulation of embryonic mesenchymal progenitor cell differentiation via control over pure mechanical modulus in electrospun nanofibers. Acta Biomaterialia.

[11] Kurtz A, Oh S-J (2012). Age related changes of the extracellular matrix and stem cell maintenance. Preventive Medicine.

[12] Bonnans C, Chou J, Werb Z (2014). Remodelling the extracellular matrix in development and disease. Nature Reviews Molecular Cell Biology.

[13] Nagase H, Visse R, Murphy G (2006). Structure and function of matrix metalloproteinases and TIMPs. Cardiovascular Research.

[14] Brizzi MF, Tarone G, Defilippi P (2012). Extracellular matrix, integrins, and growth factors as tailors of the stem cell niche. Current Opinion in Cell Biology.

[15] Kapur SK, Katz AJ (2013). Review of the adipose derived stem cell secretome. Biochimie.

[16] Salgado J (2010). A., L Reis, R., Sousa, N. & M Gimble, J. Adipose tissue derived stem cells secretome: soluble factors and their roles in regenerative medicine. Current Stem Cell Research & Therapy.

[17] Lee SC, Kim JO, Kim S-J (2015). Secretome from human adipose-derived stem cells protects mouse liver from hepatic ischemia–reperfusion injury. Surgery.

[18] Zhao L, Johnson T, Liu D (2017). Therapeutic angiogenesis of adipose-derived stem cells for ischemic diseases. Stem Cell Research & Therapy.

[19] Mazo M (2012). Treatment of reperfused ischemia with adipose-derived stem cells in a preclinical Swine model of myocardial infarction. Cell Transplantation.

[20] Thirumala S, Wu X, Gimble JM, Devireddy RV (2010). Evaluation of polyvinylpyrrolidone as a cryoprotectant for adipose tissue-derived adult stem cells. Tissue Engineering Part C: Methods.

[21] Shaik S, Hayes D, Gimble J, Devireddy R (2017). Inducing Heat Shock Proteins Enhances the Stemness of Frozen–Thawed Adipose Tissue-Derived Stem Cells. Stem Cells and Development.

[22] Shaik S, Wu X, Gimble J, Devireddy R (2018). Effects of Decade Long Freezing Storage on Adipose Derived Stem Cells Functionality. Scientific Reports.

[23] Thirumala S, Gimble JM, Devireddy RV (2010). Cryopreservation of stromal vascular fraction of adipose tissue in a serum‐free freezing medium. Journal of Tissue Engineering and Regenerative Medicine.

[24] Livak KJ, Schmittgen TD (2001). Analysis of relative gene expression data using real-time quantitative PCR and the 2− ΔΔCT method. Methods.

[25] Huang DW, Sherman BT, Lempicki RA (2009). Systematic and integrative analysis of large gene lists using DAVID bioinformatics resources. Nature Protocols.

[26] Robinson JT (2011). Integrative genomics viewer. Nature Biotechnology.

[27] Huang DW, Sherman BT, Lempicki RA (2008). Bioinformatics enrichment tools: paths toward the comprehensive functional analysis of large gene lists. Nucleic Acids Research.

[28] De Arcangelis A (2001). Overexpression of laminin α1 chain in colonic cancer cells induces an increase in tumor growth. International Journal of Cancer.

[29] Barczyk M, Carracedo S, Gullberg D (2010). Integrins. Cell and Tissue Research.

[30] Campbell, I. D. & Humphries, M. J. Integrin structure, activation, and interactions. *Cold Spring Harbor Perspectives in Biology*, a004994 (2011). 10.1101/cshperspect.a004994 PMC3039929 21421922

[31] Smith LR, Cho S, Discher DE (2017). Stem cell differentiation is regulated by extracellular matrix mechanics. Physiology.

[32] Ugawa Y (2017). Rho‐kinase regulates extracellular matrix‐mediated osteogenic differentiation of periodontal ligament cells. Cell Biology International.

[33] Infante A, Rodríguez CI (2018). Secretome analysis of *in vitro* aged human mesenchymal stem cells reveals IGFBP7 as a putative factor for promoting osteogenesis. Scientific Reports.

[34] Ragelle H (2017). Comprehensive proteomic characterization of stem cell-derived extracellular matrices. Biomaterials.

[35] Izu Y (2011). Type XII collagen regulates osteoblast polarity and communication during bone formation. The Journal of Cell Biology.

[36] Wang W (2012). Collagen XXIV (Col24α1) promotes osteoblastic differentiation and mineralization through TGF-β/Smads signaling pathway. International Journal of Biological Sciences.

[37] Zimmermann P (2008). Correlation of COL10A1 induction during chondrogenesis of mesenchymal stem cells with demethylation of two CpG sites in the COL10A1 promoter. Arthritis & Rheumatism.

[38] Gu J (2014). Identification and characterization of the novel Col10a1 regulatory mechanism during chondrocyte hypertrophic differentiation. Cell Death & Disease.

[39] Xu, J. & Mosher, D. Fibronectin and other adhesive Glycoproteins in The extracellular matrix: an overview (Ed: Mecham, R. P.) 41–75 (Springer, 2011).

[40] Kibbey MC, Grant DS, Kleinman HK (1992). Role of the SIKVAV site of laminin in promotion of angiogenesis and tumor growth: an *in vivo* Matrigel model. JNCI: Journal of the National Cancer Institute.

[41] Kuratomi Y (1999). Identification of metastasis-promoting sequences in the mouse laminin α1 chain. Experimental Cell Research.

[42] Mochizuki M (2007). Angiogenic activitiy of syndecan-binding laminin peptide AG73 (RKRLQVQLSIRT). Archives of Biochemistry and Biophysics.

[43] Edwards MM (2010). Mutations in Lama1 disrupt retinal vascular development and inner limiting membrane formation. Journal of Biological Chemistry.

[44] Gonzales M (2001). Structure and function of a vimentin-associated matrix adhesion in endothelial cells. Molecular Biology of the Cell.

[45] Thyboll J (2002). Deletion of the laminin α4 chain leads to impaired microvessel maturation. Molecular and Cellular Biology.

[46] Gonzalez AM (2002). Complex interactions between the laminin α4 subunit and integrins regulate endothelial cell behavior *in vitro* and angiogenesis *in vivo*. Proceedings of the National Academy of Sciences.

[47] Holmberg J, Durbeej M (2013). Laminin-211 in skeletal muscle function. Cell adhesion & Migration.

[48] Marinkovich MP (2007). Laminin 332 in squamous-cell carcinoma. Nature Reviews Cancer.

[49] Cooley MA (2014). Fibulin-1 is required for bone formation and Bmp-2-mediated induction of Osterix. Bone.

[50] Cooley MA (2008). Fibulin-1 is required for morphogenesis of neural crest-derived structures. Developmental Biology.

[51] Noda K, Nakamura T, Komatsu Y (2015). Fibulin-5 deficiency causes developmental defect of premaxillary bone in mice. Biochemical and Biophysical Research Communications.

[52] Yanagishita M (1993). Function of proteoglycans in the extracellular matrix. Pathology International.

[53] Tanaka K-i (2012). Role of osteoglycin in the linkage between muscle and bone. Journal of Biological Chemistry.

[54] Salo T, Mäkelä M, Kylmäniemi M, Autio-Harmainen H, Larjava H (1994). Expression of matrix metalloproteinase-2 and-9 during early human wound healing. Laboratory Investigation.

[55] Duerr S, Stremme S, Soeder S, Bau B, Aigner T (2004). MMP-2/gelatinase A is a gene product of human adult articular chondrocytes and is increased in osteoarthritic cartilage. Clinical and Experimental Rheumatology.

[56] Lohi J, Wilson CL, Roby JD, Parks WC (2001). Epilysin, a novel human matrix metalloproteinase (MMP-28) expressed in testis and keratinocytes and in response to injury. Journal of Biological Chemistry.

[57] Xiong D-H (2009). Genome-wide association and follow-up replication studies identified ADAMTS18 and TGFBR3 as bone mass candidate genes in different ethnic groups. The American Journal of Human Genetics.

[58] Porter S (2006). ADAMTS8 and ADAMTS15 expression predicts survival in human breast carcinoma. International Journal of Cancer.

[59] Zheng XL (2015). ADAMTS13 and von Willebrand factor in thrombotic thrombocytopenic purpura. Annual Review of Medicine.

[60] Strieter RM, Burdick MD, Gomperts BN, Belperio JA, Keane MP (2005). CXC chemokines in angiogenesis. Cytokine & Growth Factor Reviews.

[61] Belperio JA (2000). CXC chemokines in angiogenesis. Journal of Leukocyte Biology.

[62] Miyake M, Goodison S, Urquidi V, Giacoia EG, Rosser CJ (2013). Expression of CXCL1 in human endothelial cells induces angiogenesis through the CXCR2 receptor and the ERK1/2 and EGF pathways. Laboratory Investigation.

[63] Mehrad B, Keane MP, Strieter RM (2007). Chemokines as mediators of angiogenesis. Thrombosis and Haemostasis-Stuttgart.

[64] Arenberg DA (1998). Epithelial-neutrophil activating peptide (ENA-78) is an important angiogenic factor in non-small cell lung cancer. Journal of Clinical Investigation.

[65] Kim S-W (2012). Mesenchymal stem cells overexpressing GCP-2 improve heart function through enhanced angiogenic properties in a myocardial infarction model. Cardiovascular Research.

[66] Torán JL (2017). CXCL6 is an important paracrine factor in the pro-angiogenic human cardiac progenitor-like cell secretome. Scientific Reports.

[67] James, A. W. Review of signaling pathways governing MSC osteogenic and adipogenic differentiation. *Scientifica* **2013**, 17 pages (2013). 10.1155/2013/684736 PMC3874981 24416618

[68] Ling L, Nurcombe V, Cool SM (2009). Wnt signaling controls the fate of mesenchymal stem cells. Gene.

[69] Yavropoulou MP, Yovos JG (2007). The role of the Wnt signaling pathway in osteoblast commitment and differentiation. Hormones-athens.

[70] Shen B (2010). The role of BMP‐7 in chondrogenic and osteogenic differentiation of human bone marrow multipotent mesenchymal stromal cells *in vitro*. Journal of Cellular Biochemistry.

[71] Martin EC (2018). Trauma induced heterotopic ossification patient serum alters mitogen activated protein kinase signaling in adipose stem cells. Journal of Cellular Physiology.

[72] Kim JM (2013). Comparative secretome analysis of human bone marrow‐derived mesenchymal stem cells during osteogenesis. Journal of Cellular Physiology.

