# Supplementary material for: Transcriptomic Profiling of Adipose Derived Stem Cells Undergoing Osteogenesis by RNA-Seq
Source: Sci Rep. 2019 Aug 13;9:11800. doi: 10.1038/s41598-019-48089-1 (PMC6692320; doi:10.1038/s41598-019-48089-1)
Supplement: Supplementary file 1 — Supplementary Information [file 41598_2019_48089_MOESM1_ESM.pdf]

Supplemental Information

Transcriptomic Profiling of Adipose Derived Stem Cells Undergoing Osteogenesis by RNA-Seq

Shahensha Shaik[1], Elizabeth Martin[2], Daniel Hayes[3], Jeffrey Gimble[4] & Ram Devireddy[1*]

[1]Bioengineering Laboratory, Department of Mechanical Engineering,
Louisiana State University, Baton Rouge, LA.

[2]Biological & Agricultural Engineering,
Louisiana State University, Baton Rouge, LA.

[3]Department of Biomedical Engineering,
Pennsylvania State University, University Park, PA.

[4]La Cell LLC and Center for Stem Cell Research & Regenerative Medicine and Departments of Medicine, Structural & Cellular Biology, and Surgery, Tulane University School of Medicine, New Orleans, LA.

[*]To whom correspondence should be addressed.

Department of Mechanical Engineering,

Louisiana State University, Baton Rouge, LA, USA 70803

Tel: (225) 578-5891; Fax: (225) 578-5924;

E-mail: rdevir1@me.lsu.edu

Alizarin Red Staining-21 days

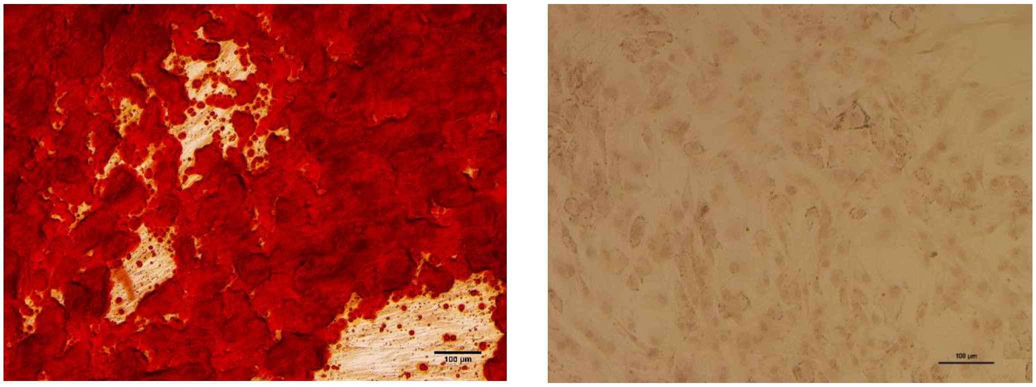

Figure S1:

Osteogenic differentiation induction after 21 days, **Fig 1A (Left Image):** Alizarin Red S staining of ASCs 21 days after osteogenic induction. **Fig. 1B (Right Image)** Alizarin Red S staining of undifferentiated ASCs after 21 days of culture. Image magnification size of 10x with scale bar of 100 μm (in both the figures)

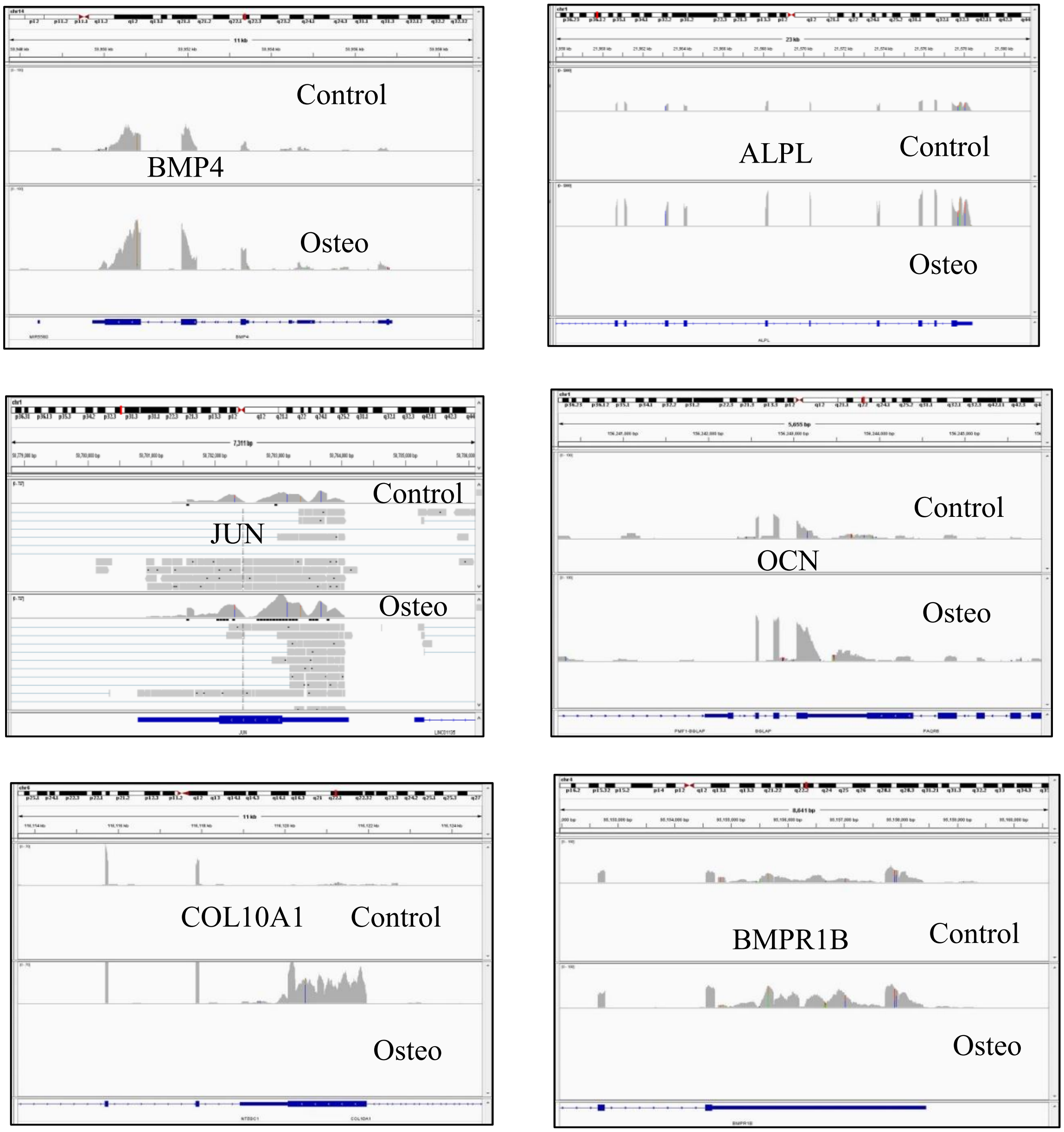

Figure S2:

Representation of upregulation of osteogenic genes (ALPL, BMP4, BMPR1B, COL10A1, JUN, and OCN) as visualized in integrative genomics viewer after alignment of with human genome HG38.

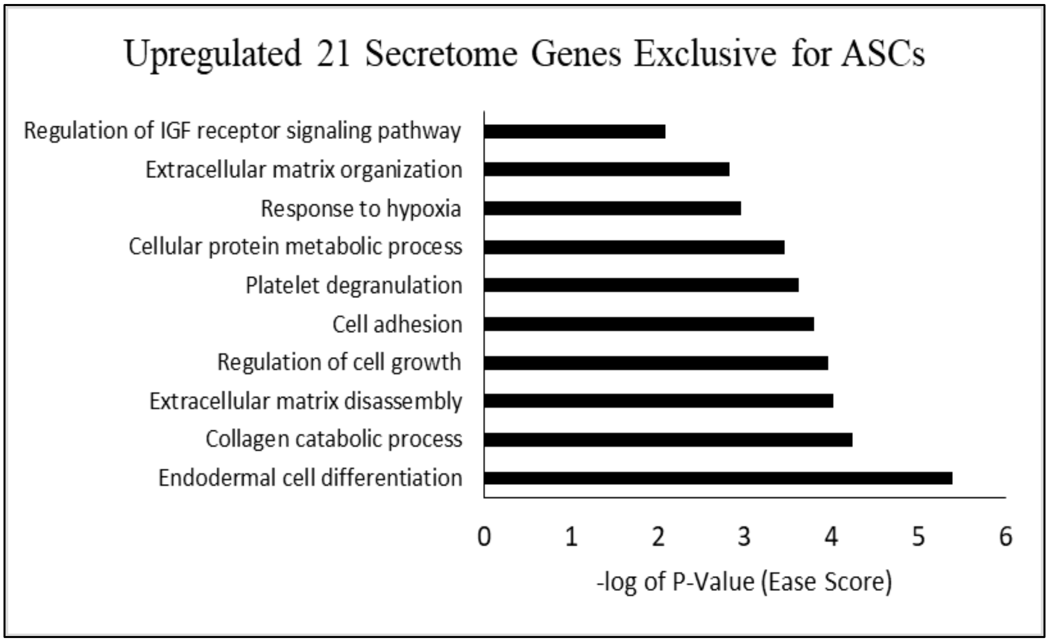

Figure S3:

Gene ontology functional analysis of 21 secretome genes upregulated exclusively in ASCs under biological process category.

Supplemental Tables:

Table S1: Primer Sequences

Gene	Gene Forward (5'-3')	Reverse (5'-3')
ANGPT1	GCAGAGCAGACCAGAAAG	CTCTAGCTTGTAGGTGGATAATG
HGF	GGGAACCAGATGCAAGTAAG	CCCAAGGAATGAGTGGATTT
CCL2	CGCGAGCTATAGAAGAATCAC	GAATCCTGAACCCACTTCTG
CXCL1	GCCCAAACCGAAGTCATAG	TTGTCACTGTTCAGCATCTT
CXCL5	GCAAGGAGTTCATCCCAAA	ATTTCCTTCCCGTTCTTCAG
CXCL6	CTGCGTTGCACTTGTTTAC	ACTTGCTTCCCGTTCTTC
CXCL8	TTGCCAAGGAGTGCTAAAG	CACTCTCAATCACTCTCAGTTC
MMP16	ACAGGGCATCCAGAAGATA	GTCAGCCGGAGGAATAGA
MMP1	CTGACATTCACCAAGGTCTC	TTCCTCCAGGTCCATCAA
MMP3	GCCAGGGATTAATGGAGATG	TGAGCAGCAACGAGAAATAA
ADAMTS6	GGCCACCCTCATGTTATTT	CCAAGGTTTGCCACTTCT
ADAMTS14	GAGGAGATGGACACCTATGA	AGCCGTATTTGGTGAACTG
GAPDH	GGTGTGAACCATGAGAAGTATGA	GAGTCCTTCCACGATACCAAAG
LAMA4	CCGACTGGTAATTGATGGTCTC	GAGCCACACCTCCCAAATAA
LAMB1	CAGCAGCCGATGTGGTAATA	CCGTGTAGTTTGTTCCCTTCT
WNT10B	TGACATGGACTTTGGAGAGAAG	GTTGTGGATTCGCATTCGTG
FOXF1	CCGAAAGGAGTTTGTCTTCTCT	AAGGCTTGATGTCTTGGTAGG
FBLN1	TCAACACAGTGGGCTCTTTC	CCACTCTCACACTCGTCAATATC
FERMT1	CCTCGGTCTCTGGTTGATAAAG	CATCCTCTTGGATGCCTTGT
IGF1	GGAGGCTGGAGATGTATTGC	ACTTCCTTCTGGGTCTTGGG
Col10A1	ACCCAAGGACTGGAATCTTTAC	GCCATTCTTATACAGGCCTACC
Col11A1	GGAGAGAAAGGACAGAAAGGAG	AGACCTGGAGGACCCATAATA
FN1	CCACAGTGGAGTATGTGGTTAG	CAGTCCTTTAGGGCGATCAAT

Table S2: Osteogenic genes expression during Osteogenesis. Genes above 1.5 FC are upregulated; between 1.49-0.67 FC are unchanged; below 0.66 FC are downregulated

Gene Name	Control Count (CC)	Control FPKM	Osteo Count (OC)	Osteo FPKM	Fold change (OC/CC)
UPREGULATED					
COL10A1	5.00	0.05	281.00	3.65	56.20
NOG	2.00	0.03	91.04	1.25	45.52
CTSK	3045.95	50.63	71657.01	1164.82	23.53
VCAM1	75.00	0.62	801.89	6.58	10.69
ICAM1	1376.00	13.54	6718.76	62.22	4.88
CSF1	5126.15	39.96	17666.67	165.43	3.45
BGLAP	1.02	0.03	3.15	0.10	3.09
ALPL	6860.62	71.52	20461.99	207.68	2.98
VEGFB	1231.00	39.85	3432.78	102.80	2.79
TGFBR2	14775.10	79.97	41096.61	220.77	2.78
FN1	1222356.30	4839.91	3391540.64	12484.05	2.77
IGF1	50.92	0.72	137.92	3.30	2.71
TNF	2.00	0.03	5.00	0.08	2.50
TWIST1	347.99	10.36	753.89	22.50	2.17
EGFR	5323.00	22.15	9910.34	40.93	1.86
BMPR1B	214.00	1.24	386.00	2.71	1.80
MMP2	84805.54	1162.58	146515.17	1924.06	1.73
ITGA3	1370.39	8.67	2189.73	17.48	1.60
BMP4	91.00	1.69	144.00	2.88	1.58
UNCHANGED					
TGFB2	80.00	0.83	118.60	0.58	1.48
FGF1	515.00	9.37	729.98	9.11	1.42
SMAD2	2143.02	28.33	2968.44	43.56	1.39
VDR	3100.04	22.77	4255.75	31.69	1.37
CDH11	21039.55	251.55	27769.99	338.96	1.32
RUNX2	799.00	4.72	977.15	7.36	1.22
IGF1R	1201.78	7.10	1409.93	7.15	1.17
IGF2	595.84	12.25	699.02	11.73	1.17
SMAD5	2587.75	47.31	2980.82	54.34	1.15
BMPR1A	1390.97	5.63	1582.99	7.15	1.14
SMAD4	2826.03	42.65	3029.22	41.46	1.07
NFKB1	2743.51	20.78	2924.19	20.19	1.07
BMPR2	2208.98	4.77	2333.91	4.96	1.06
ANXA5	50766.40	787.14	52595.98	814.51	1.04
FGFR1	9531.37	90.64	9532.73	91.58	1.00
CSF3	3.00	0.17	3.00	0.10	1.00

Gene Name	Control Count (CC)	Control FPKM	Osteo Count (OC)	Osteo FPKM	Fold change (OC/CC)
SMAD3	5425.58	39.72	5155.08	37.06	0.95
ACVR1	3542.93	33.94	3043.30	29.04	0.86
ITGA1	1696.81	9.47	1357.58	9.10	0.80
ITGB1	73720.66	818.24	56204.84	582.55	0.76
TGFBR1	4759.46	41.74	3530.18	34.88	0.74
FGFR2	33.01	0.23	22.00	0.25	0.67
DOWNREGULATED					
BMP6	366.00	3.37	237.92	2.17	0.65
COL14A1	1146.51	6.10	702.44	3.50	0.61
SMAD1	1006.97	21.72	554.94	11.09	0.55
CD36	4527.04	71.63	2179.55	37.17	0.48
COL2A1	11.72	0.15	5.54	0.04	0.47
TGFB1	4702.29	71.86	2142.55	37.60	0.46
BGN	31704.07	355.83	14372.98	159.78	0.45
ITGA2	530.08	2.42	231.79	1.11	0.44
SERPINH1	50230.14	594.67	20322.02	240.22	0.40
SOX9	159.00	1.02	63.00	0.40	0.40
TGFB3	2967.49	46.23	1126.39	17.51	0.38
TNFSF11	2.00	0.02	0.75	0.02	0.38
VEGFA	6793.53	101.33	2374.33	42.43	0.35
BMP1	11008.33	91.64	3746.41	28.19	0.34
CHRD	359.99	2.85	119.68	1.13	0.33
COMP	29490.45	319.44	8202.31	88.03	0.28
COL1A2	1971876.43	10568.52	528408.51	2945.91	0.27
FLT1	314.23	2.35	76.94	0.48	0.24
FGF2	5042.52	25.38	1172.12	6.17	0.23
GLI1	33.00	0.62	7.00	0.54	0.21
MMP9	139.05	1.54	23.00	0.25	0.17
COL15A1	36604.01	168.56	4998.10	22.96	0.14
COL3A1	947109.50	4643.73	128696.18	629.60	0.14
COL5A1	91669.91	309.53	12438.10	47.76	0.14
PDGFA	251.00	6.46	33.00	0.77	0.13
SPP1	198.07	4.08	25.00	0.82	0.13
COL1A1	2797698.02	15091.95	341861.76	2045.01	0.12
BMP2	109.83	0.77	11.00	0.08	0.10
PHEX	73.00	0.29	4.00	0.03	0.05
GDF10	433.96	4.55	10.24	0.11	0.02
BMP3	3.00	0.01	0.00	0.00	0.00
BMP7	1.00	0.02	0.00	0.00	0.00
CALCR	1.00	0.01	0.00	0.00	0.00
DLX5	1.01	0.03	0.00	0.00	0.00

Gene Name	Control Count (CC)	Control FPKM	Osteo Count (OC)	Osteo FPKM	Fold change (OC/CC)
EGF	3.00	0.06	0.00	0.00	0.00
MMP10	30.04	0.45	0.00	0.00	0.00

Table S3: Glycoproteins expression during Osteogenesis. Genes above 1.5 FC are upregulated; between 1.49-0.67 FC are unchanged; below 0.66 FC are downregulated.

Gene	Control Count (CC)	Control FPKM	Osteo Count (OC)	Osteo FPKM	Fold Change (OC/CC)
UPREGULATED					
PAPLN	39.00	0.50	1481.80	11.46	38.00
LAMA1	567.40	2.40	7645.00	25.56	13.47
DPT	2065.70	30.60	18033.20	263.90	8.73
FRAS1	129.00	0.33	1077.10	2.30	8.35
LAMA2	1346.00	4.67	7654.10	19.50	5.69
LTBP1	2924.00	19.70	14915.10	108.48	5.10
NPNT	25.00	0.47	125.00	1.34	5.00
LAMA3	93.00	0.70	426.00	1.89	4.58
VWA5A	343.00	3.10	1372.20	11.73	4.00
FBLN1	37245.60	392.60	130866.10	1417.66	3.51
NID1	11359.90	48.10	39623.40	166.46	3.49
DMBT1	7.00	0.00	24.40	0.09	3.48
SRPX2	1551.40	19.70	4733.70	60.23	3.05
MFAP4	6489.80	92.10	18101.20	255.86	2.79
FN1	1222356.00	4839.90	3391541.00	12484.05	2.77
LAMA4	14447.70	90.30	37124.60	234.01	2.57
VWF	635.00	2.80	1513.00	8.21	2.38
FBLN5	7990.70	124.50	17553.00	269.13	2.20
THBS1	72760.70	351.30	159591.00	873.93	2.19
IGFBP7	10382.50	225.70	21559.10	454.89	2.08
BMPER	850.00	8.30	1573.30	15.80	1.85
**UNCHANGED**					
EMILIN1	8097.90	62.60	11758.20	87.65	1.45
MFAP5	13652.20	434.30	18881.60	626.48	1.38
MFGE8	16712.10	250.60	22078.60	333.86	1.32
LAMC1	86772.70	304.40	111289.70	411.13	1.28
MFAP2	5265.90	138.60	6525.40	167.11	1.24
EFEMP2	15612.20	238.00	17559.60	270.50	1.12
LTBP4	704.40	13.50	745.10	17.42	1.06
ECM1	6902.50	85.90	5985.70	73.80	0.87
LAMB2	32170.30	382.90	25832.10	347.23	0.80
PXDN	32664.00	160.10	26129.00	119.41	0.80
LTBP2	28418.40	94.80	22669.10	77.21	0.80
EMILIN2	8078.50	35.90	6252.90	28.41	0.77

Gene	Control Count (CC)	Control FPKM	Osteo Count (OC)	Osteo FPKM	Fold Change (OC/CC)
THBS4	39.01	0.30	30.00	0.63	0.77
COLQ	23.74	0.40	16.90	0.37	0.71
EFEMP1	71278.10	972.40	50595.30	679.25	0.71
LAMA5	788.30	4.20	548.80	3.29	0.70
LAMC2	1446.30	8.50	971.60	5.02	0.67
**DOWNREGULATED**					
ABI3BP	14098.40	192.10	8970.00	141.05	0.64
FBN1	70753.10	231.20	40413.60	129.62	0.57
FBLN2	92585.00	622.80	52600.90	339.54	0.57
MATN2	782.00	6.10	411.60	3.16	0.53
LAMB3	983.00	6.90	464.00	3.95	0.47
TINAGL1	2209.90	39.30	985.90	16.33	0.45
AGRN	586.30	4.20	256.40	1.85	0.44
HMCN1	892.01	3.90	385.90	1.94	0.43
MMRN2	405.60	4.50	157.60	1.85	0.39
PCOLCE2	1874.00	35.00	654.00	12.57	0.35
EMID1	3.00	0.00	1.00	0.03	0.33
VWA1	324.00	4.10	102.20	1.39	0.32
SPON1	4593.00	24.60	1251.00	6.40	0.27
TNXB	2197.60	11.10	501.60	2.14	0.23
TGFBI	258297.30	2882.60	40683.90	425.50	0.16
POSTN	81743.90	654.50	12700.70	101.66	0.16
NTN1	37.00	0.20	5.00	0.02	0.14
ELN	87555.30	950.80	5366.60	59.98	0.06
VTN	184.00	2.40	8.00	0.10	0.04
TNC	89478.40	453.80	2757.50	13.62	0.03
HMCN2	7.00	0.00	0.00	0.00	0.00

Table S4: Collagens expression during Osteogenesis. Genes above 1.5 FC are upregulated; between 1.49-0.67 FC are unchanged; below 0.66 FC are downregulated.

Gene	Control Count (CC)	Control FPKM	Osteo Count (OC)	Osteo FPKM	Fold Change (OC/CC)
UPREGULATED					
COL10A1	5.00	0.05	281.00	3.65	56.20
COL4A4	171.00	0.42	1518.39	3.74	8.88
COL4A5	55.53	0.38	362.00	1.94	6.52
COL24A1	3.00	0.01	17.00	0.06	5.67
COL4A6	2.11	0.01	10.00	0.04	4.74
COL12A1	15090.88	57.83	23514.44	84.27	1.56
UNCHANGED					
COL25A1	4.00	0.03	5.96	0.06	1.49
COL6A3	189668.70	567.89	222463.10	660.52	1.17
COL23A1	3.00	0.03	3.00	0.03	1.00
COL6A2	223599.50	1836.67	182533.30	1514.66	0.82
COL27A1	163.30	0.79	126.00	0.70	0.77
COL9A3	12.00	0.29	9.04	0.16	0.75
COL4A2	44609.36	226.60	30460.99	148.59	0.68
DOWNREGULATED					
COL16A1	22096.57	165.66	14218.44	105.22	0.64
COL14A1	1146.51	6.10	702.44	3.50	0.61
COL6A1	203295.20	1503.64	122988.60	893.04	0.60
COL18A1	8870.32	43.84	4349.17	21.33	0.49
COL4A1	32614.27	156.72	13532.08	61.41	0.41
COL13A1	279.00	2.97	94.03	1.42	0.34
COL5A2	46475.27	183.13	15574.58	66.76	0.34
COL1A2	1971876.00	10568.52	528408.50	2945.91	0.27
COL7A1	772.58	3.77	193.88	1.30	0.25
COL15A1	36604.01	168.56	4998.10	22.96	0.14
COL3A1	947109.50	4643.73	128696.20	629.60	0.14
COL5A1	91669.91	309.53	12438.10	47.76	0.14
COL1A1	2797698.00	15091.95	341861.80	2045.01	0.12
COL5A3	8474.96	34.40	690.81	2.89	0.08

Table S5: **Proteoglycans expression during Osteogenesis. Genes above 1.5 FC are upregulated; between 1.49-0.67 FC are unchanged; below 0.66 FC are downregulated.**

Gene	Control Count (CC)	Control FPKM	Osteo Count (OC)	Osteo FPKM	Fold Change (OC/CC)
UPREGULATED					
HAPLN1	3	0.03	45	0.48	15
OGN	38	0.35	108	1.37	2.84
UNCHANGED					
DCN	65262.77	1172.39	85101.73	1691.45	1.3
LUM	12226.75	267.15	15030.53	331.05	1.22
HSPG2	8218.38	37.32	8168.33	39.35	0.99
ASPN	1493.97	16.02	1439.05	15.36	0.96
VCAN	21997.59	102.3	15834.76	75.9	0.71
DOWNREGULATED					
PRELP	3606.78	15.57	2264.59	9.71	0.62
BGN	31704.07	355.83	14372.98	159.78	0.45

Table S6: **ECM Affiliated Proteins gene expression during Osteogenesis. Genes above 1.5 FC are upregulated; between 1.49-0.67 FC are unchanged; below 0.66 FC are downregulated**

Gene	Control Count (CC)	Control FPKM	Osteo Count (OC)	Osteo FPKM	Fold Change (OC/CC)
UPREGULATED					
PLXDC2	512.00	3.99	2142.67	16.37	4.18
ANXA9	2.00	0.03	8.00	0.11	4.00
LGALS3	6197.81	214.73	18369.56	607.02	2.96
ANXA6	9678.46	107.23	20096.99	238.01	2.08
FREM1	6.00	0.07	10.00	0.09	1.67
COLEC12	2221.69	10.41	3683.47	17.57	1.66
SFTPB	2.00	0.01	3.00	0.24	1.50
UNCHANGED					
CLEC14A	151.00	1.73	216.75	2.45	1.44
ANXA2	145163.00	2810.60	155832.00	2967.33	1.07
ANXA5	50766.40	787.14	52595.98	814.51	1.04
ANXA11	14312.73	145.57	12970.12	126.46	0.91
SEMA3C	4507.95	46.78	3938.97	42.82	0.87
ANXA7	10304.57	166.44	8986.09	143.99	0.87
ANXA1	21008.85	385.40	17641.95	318.63	0.84
PLXNB2	14783.69	102.32	11672.08	81.21	0.79
DOWNREGULATED					
LGALS4	2.00	0.06	1.00	0.09	0.50
CSPG4	3712.72	11.02	932.44	2.75	0.25

Table S7: ECM Regulators gene expression during Osteogenesis. Genes above 1.5 FC are upregulated; between 1.49-0.67 FC are unchanged; below 0.66 FC are downregulated

Gene	Control Count (CC)	Control FPKM	Osteo Count (OC)	Osteo FPKM	Fold change (OC/CC)
UPREGULATED					
SERPINF2	4.00	0.05	46.00	0.52	11.50
ITIH3	9.00	0.28	34.00	1.11	3.78
CTSB	91894.27	1282.22	282894.50	3819.71	3.08
ITIH4	2.00	0.07	4.66	0.26	2.33
SERPINC1	1.00	0.02	2.00	0.05	2.00
HTRA1	41243.08	696.12	78015.36	1305.63	1.89
ELANE	22.00	0.66	36.00	1.09	1.64
SERPING1	25134.88	423.88	38369.54	639.03	1.53
UNCHANGED					
ADAMTSL1	5079.73	33.26	7503.98	52.17	1.48
ITIH1	1.00	0.04	0.99	0.04	0.99
ADAMTSL5	197.60	2.48	184.00	1.97	0.93
PLOD1	21992.63	217.23	19792.78	191.08	0.90
ADAM10	6269.69	91.70	5447.79	78.84	0.87
LOXL1	8881.70	282.89	6660.64	211.56	0.75
PLOD3	9786.94	112.18	7057.29	84.66	0.72
DOWNREGULATED					
ITIH5	2169.99	15.66	1038.75	6.32	0.48
SERPINH1	50230.14	594.67	20322.02	240.22	0.40
ADAMTS17	35.04	0.59	12.27	0.26	0.35
LOX	67364.79	549.12	23487.24	199.77	0.35
TGM2	5370.16	35.56	1246.03	7.57	0.23
CPN2	11.00	0.12	2.00	0.03	0.18
MMP9	139.05	1.54	23.00	0.25	0.17
ADAM19	4360.31	21.27	411.77	1.97	0.09
AMBP	2.00	0.04	0.00	0.00	0.00
F13A1	6.00	0.04	0.00	0.00	0.00
PLG	12.99	0.62	0.00	0.00	0.00
TGM3	1.00	0.01	0.00	0.00	0.00

Table S8: Secreted factors gene expression during Osteogenesis. Genes above 1.5 FC are upregulated; between 1.49-0.67 FC are unchanged; below 0.66 FC are downregulated

Gene	Control Count (CC)	Control FPKM	Osteo Count (OC)	Osteo FPKM	Fold Change (OC/CC)
UPREGULATED					
PF4	6	0.32	30	1.57	5
IL16	98.34	0.67	401.88	2.25	4.08
S100A13	4311.37	215.58	9683.15	487.87	2.24
UNCHANGED					
HCFC1	1787.63	6.5	1641.14	5.62	0.91
S100A11	36456.17	2000.03	32029.3	1711.62	0.87
DOWNREGULATED					
EGFL7	503.97	11.31	171.31	3.84	0.33

Table S9: Matrix metalloproteinases (MMPs) gene expression during Osteogenesis. Genes above 1.5 FC are upregulated; between 1.49-0.67 FC are unchanged; below 0.66 FC are downregulated

Gene Name	Control Count (CC)	Control FPKM	Osteo Count (OC)	Osteo FPKM	Fold change (OC/CC)
UPREGULATED					
MMP2	84805.54	1162.58	146515.2	1924.06	1.72766
MMP15	88	0.75	141.56	0.96	1.608636
MMP28	15	0.3	23	0.45	1.533333
UNCHANGED					
MMP17	487	10.47	684.01	15.98	1.404538
MMP14	5098.83	35.53	6874.55	48.37	1.34826
MMP19	1471.09	17.04	1602.39	17.84	1.089254
MMP23A	53.94	1.51	49.42	1.36	0.916203
MMP23B	52.06	2.38	45.6	2.28	0.875912
MMP25	11.4	0.37	9.52	0.14	0.835088
DOWNREGULATED					
MMP11	986	11.42	621.54	7.18	0.630365
MMP24	124.18	0.7	59.25	0.33	0.47713
MMP9	139.05	1.54	23	0.25	0.165408
MMP12	27	0.38	3	0.04	0.111111
MMP27	12	0.16	1	0.01	0.083333
MMP16	599.47	3.52	48.02	0.34	0.080104
MMP1	2327.16	30.94	64.99	0.86	0.027927
MMP3	2299.93	40.53	51	0.69	0.022175
MMP10	30.04	0.45	0	0	0

Table S10: ADAMTS gene expression during Osteogenesis. Genes above 1.5 FC are upregulated; between 1.49-0.67 FC are unchanged; below 0.66 FC are downregulated

Gene	Control Count (CC)	Control FPKM	Osteo Count (OC)	Osteo FPKM	Fold change (OC/CC)
UPREGULATED					
ADAMTS18	5	0.06	102	0.86	20.40
ADAMTS15	268	1.17	797.11	3.46	2.97
ADAMTS8	9	0.07	17	0.11	1.89
ADAMTS13	95	0.99	149.58	1.6	1.57
UNCHANGED					
ADAMTS5	643.14	1.74	957.62	2.58	1.49
ADAMTS3	13.59	0.1	10.02	0.1	0.74
DOWNREGULATED					
ADAMTS1	19055.99	116.12	12139.69	77.74	0.64
ADAMTS12	1476	7.5	904.56	5.05	0.61
ADAMTS4	2075.69	14.69	1072.02	7.35	0.52
ADAMTS7	1381.39	6.49	711.48	3.42	0.52
ADAMTS20	2	0.01	1	0.01	0.50
ADAMTS2	34612.12	150.44	14892.77	74.04	0.43
ADAMTS17	35.04	0.59	12.27	0.26	0.35
ADAMTS10	989.01	8.34	327.01	5.08	0.33
ADAMTS6	764.97	5.12	184	1.15	0.24
ADAMTS9	90	0.9	20	0.21	0.22
ADAMTS16	111	0.64	23	0.17	0.21
ADAMTS14	833.36	3.94	45.01	0.21	0.05

Table S11: Integrins α gene expression during Osteogenesis. Genes above 1.5 FC are upregulated; between 1.49-0.67 FC are unchanged; below 0.66 FC are downregulated

Gene	Control Count (CC)	Control FPKM	Osteo Count (OC)	Osteo FPKM	Fold Change (OC/CC)
UPREGULATED					
ITGA10	142.00	0.81	714.98	4.47	5.04
ITGA4	1165.02	9.07	2684.12	20.02	2.30
ITGA7	139.00	1.56	241.95	2.55	1.74
ITGAE	601.00	27.19	1003.86	44.81	1.67
ITGA3	1370.39	8.67	2189.73	17.48	1.60
UNCHANGED					
ITGA2B	3.00	0.04	4.00	0.11	1.33
ITGAV	11932.07	64.20	10562.76	56.53	0.89
ITGA11	21458.88	112.66	18406.23	102.71	0.86
ITGA1	1696.81	9.47	1357.58	9.10	0.80
DOWNREGULATED					
ITGA6	291.00	1.80	167.61	0.96	0.58
ITGA5	69376.17	444.23	32210.08	194.89	0.46
ITGA2	530.08	2.42	231.79	1.11	0.44
ITGA9	145.00	0.54	41.00	0.17	0.28
ITGA8	1629.56	5.99	208.24	0.76	0.13

Table S12: Integrins β gene expression during Osteogenesis. Genes above 1.5 FC are upregulated; between 1.49-0.67 FC are unchanged; below 0.66 FC are downregulated

Gene Name	Control Count (CC)	Control FPKM	Osteo Count (OC)	Osteo FPKM	Fold Change (OC/CC)
UPREGULATED					
ITGB2	25.66	0.61	251.35	2.82	9.80
ITGB8	51.00	0.22	430.97	1.33	8.45
ITGBL1	3934.14	40.86	11163.84	112.77	2.84
ITGB4	19.00	0.88	32.00	1.22	1.68
UNCHANGED					
ITGB5	24468.94	277.00	33227.13	397.80	1.36
ITGB1	73720.66	818.24	56204.84	582.55	0.76
DOWNREGULATED					
ITGB7	16.73	0.96	6.30	0.14	0.38
ITGB6	6.00	0.31	2.00	0.13	0.33
ITGB3	6649.56	45.58	1982.70	14.38	0.30

Table S13: Angiogenic genes expression during Osteogenesis. Genes above 1.5 FC are upregulated; between 1.49-0.67 FC are unchanged; below 0.66 FC are downregulated

Gene	Control Count (CC)	Control FPKM	Osteo Count (OC)	Osteo FPKM	Fold Change (OC/CC)
UPREGULATED					
LEP	1.00	0.01	1150.22	8.44	1150.22
ANGPT1	120.00	1.13	1639.72	12.22	13.66
HGF	68.79	0.64	935.39	8.06	13.60
CCL2	1101.39	44.86	7375.72	294.00	6.70
PF4	6.00	0.32	30.00	1.57	5.00
S1PR1	168.00	1.49	839.99	7.79	5.00
CXCL5	79.00	0.80	376.09	3.77	4.76
EDN1	55.02	0.71	235.68	3.00	4.28
SERPINF1	6771.28	145.12	28274.91	564.73	4.18
CXCL1	32.00	0.82	121.00	3.12	3.78
HPSE	33.00	0.44	103.00	1.35	3.12
VEGFB	1231.00	39.85	3432.78	102.80	2.79
FN1	1222356.00	4839.91	3391541.00	12484.05	2.77
IGF1	50.92	0.72	137.92	3.30	2.71
ANG	188.77	5.26	505.80	16.35	2.68
TNF	2.00	0.03	5.00	0.08	2.50
CXCL6	59.00	1.29	136.87	2.14	2.32
CXCL8	38.00	0.70	87.00	1.65	2.29
THBS1	72760.73	351.28	159590.70	873.93	2.19
MMP2	84805.54	1162.58	146515.20	1924.06	1.73
TIMP2	2247.62	15.61	3657.11	25.26	1.63
NRP2	2841.14	25.60	4531.49	35.59	1.59
EFNB2	50.00	0.28	77.00	0.43	1.54
UNCHANGED					
TGFB2	80.00	0.83	118.60	0.58	1.48
B2M	49015.89	2972.76	71836.89	4281.24	1.47
RPLP0	43175.18	1014.17	62092.34	1420.64	1.44
ANPEP	30978.59	229.95	44022.25	336.73	1.42
FGF1	515.00	9.37	729.98	9.11	1.42
VEGFC	780.00	9.86	1077.18	13.53	1.38
MDK	623.98	25.13	844.34	30.35	1.35
MMP14	5098.83	35.53	6874.55	48.37	1.35
HPRT1	389.00	7.50	474.12	9.02	1.22
FIGF	10.00	0.13	12.00	0.23	1.20
CDH5	420.00	2.80	502.22	3.18	1.20
ERBB2	3379.53	23.41	3982.72	30.47	1.18
CTGF	28368.05	313.50	31474.00	344.52	1.11
AKT1	8108.95	151.03	7827.79	143.68	0.97
TYMP	3365.03	52.23	3115.81	48.12	0.93

Gene	Control Count (CC)	Control FPKM	Osteo Count (OC)	Osteo FPKM	Fold Change (OC/CC)
F3	1718.92	25.53	1546.37	21.01	0.90
ITGAV	11932.07	64.20	10562.76	56.53	0.89
HIF1A	30768.83	275.48	26896.90	243.15	0.87
EPHB4	717.00	4.80	597.13	4.78	0.83
PTGS1	15247.04	104.68	12246.96	74.66	0.80
SPHK1	8347.09	122.52	6192.10	91.47	0.74
TGFBR1	4759.46	41.74	3530.18	34.88	0.74
NOTCH4	180.02	1.33	131.72	1.11	0.73
THBS2	109805.50	561.15	76932.39	408.36	0.70
DOWNREGULATED					
NRP1	23071.50	126.45	14832.16	87.05	0.64
CCL11	26.00	0.68	16.00	0.41	0.62
ANGPTL4	3328.63	48.22	1931.33	29.18	0.58
JAG1	3648.70	18.55	2066.65	11.21	0.57
PECAM1	1258.00	11.25	691.78	7.92	0.55
NOS3	105.00	1.56	55.62	1.42	0.53
TIE1	259.46	2.67	136.00	1.10	0.52
CXCL10	6.00	0.14	3.00	0.07	0.50
COL18A1	8870.32	43.84	4349.17	21.33	0.49
TEK	1268.01	6.83	595.41	3.17	0.47
PLAU	6345.83	70.61	2953.66	33.18	0.47
TGFB1	4702.29	71.86	2142.55	37.60	0.46
TIMP1	344201.20	12980.09	155370.50	5671.80	0.45
EFNA1	68.00	1.74	29.00	0.66	0.43
IL6	775.33	30.83	301.88	10.58	0.39
VEGFA	6793.53	101.33	2374.33	42.43	0.35
ID1	1173.01	33.00	404.86	11.31	0.35
TGFA	6.00	0.04	2.00	0.01	0.33
ITGB3	6649.56	45.58	1982.70	14.38	0.30
KDR	315.00	1.34	89.98	0.45	0.29
ENG	34690.63	299.68	9584.04	80.93	0.28
ANGPT2	878.08	8.23	230.99	2.15	0.26
FLT1	314.23	2.35	76.94	0.48	0.24
FGF2	5042.52	25.38	1172.12	6.17	0.23
TIMP3	229675.60	1246.62	49771.83	268.16	0.22
FGFR3	76.00	0.45	14.00	0.08	0.18
IL1B	67.00	1.34	12.17	0.20	0.18
MMP9	139.05	1.54	23.00	0.25	0.17
SERPINE1	592319.90	4711.14	89094.11	702.74	0.15
PDGFA	251.00	6.46	33.00	0.77	0.13
PGF	1191.01	18.41	83.00	1.27	0.07
ADGRB1	1.00	0.01	0.00	0.00	0.00
EGF	3.00	0.06	0.00	0.00	0.00
IFNA1	14.55	0.50	0.00	0.00	0.00

Gene	Control Count (CC)	Control FPKM	Osteo Count (OC)	Osteo FPKM	Fold Change (OC/CC)
PLG	12.99	0.62	0.00	0.00	0.00
